# Parthanatos initiated by ROS-induced DNA damage is involved in intestinal epithelial injury during necrotizing enterocolitis

**DOI:** 10.1038/s41420-024-02114-z

**Published:** 2024-07-31

**Authors:** Lingqi Xu, Shurong Ma, Minhan Qu, Na Li, Xu Sun, Tingting Wang, Lulu Chen, Jie Zhu, Yifang Ding, Yuan Gong, Fangjie Hu, Zhenzhen Dong, Rui Zhang, Jiang Huai Wang, Jian Wang, Huiting Zhou

**Affiliations:** 1 Institute of Pediatric Research, Children’s Hospital of Soochow University, Suzhou, China; 2 Department of Surgery, Children’s Hospital of Soochow University, Suzhou, China; 3 Department of Pediatrics, The Affiliated Zhangjiagang Hospital of Soochow University, Suzhou, China; 4 Department of Academic Surgery, University College Cork, Cork University Hospital, Cork, Ireland

**Keywords:** Cell death, Infant necrotizing enterocolitis

## Abstract

Necrotizing enterocolitis (NEC) involves intestinal epithelial damage and inflammatory response and is associated with high morbidity and mortality in infants. To improve therapeutic prospects, elucidating underlying molecular mechanisms of intestinal epithelial damage during NEC is of the essence. Poly (ADP-ribose) polymerase 1 (PARP1)-dependent parthanatos is a programmed inflammatory cell death. In the present study, the presence of parthanatos-associated proteins PARP1 and poly (ADP-ribose) (PAR), along with high expression of DNA damage-associated biomarkers, 8-hydroxy-2’-deoxyguanosine (8-OHdG) and phosphorylation of histone H2AX (γH2AX), were discovered in the intestinal tissues of NEC infants. Additionally, the upregulated expression of PARP1 and PAR in NEC intestinal tissues correlated distinctly with clinical indices indicative of NEC incidence and severity. Furthermore, we demonstrated that inhibiting the expression of parthanatos-associated proteins, by either pharmacological blockage using 3-aminobenzamide (3-AB), an inhibitor of PARP1, or genetic knockout using *Parp1*-deficient mice, resulted in substantial improvements in both histopathological severity scores associated with intestinal injury and inflammatory reactions. Moreover, in an in vitro NEC model, reactive oxygen species (ROS)-induced DNA damage promoted the formation of PAR and nuclear translocation of apoptosis-inducing factor (AIF), thus activating PARP1-dependent parthanatos in Caco-2 cells and human intestinal organoids. Our work verifies a previously unexplored role for parthanatos in intestinal epithelial damage during NEC and suggests that inhibition of parthanatos may serve as a potential therapeutic strategy for intervention of NEC.

## Introduction

Neonatal necrotizing enterocolitis (NEC) is the most common life-threatening acute gastrointestinal inflammatory disease characterized by an excessive inflammatory response and intestinal epithelial injury [1], typically seen in preterm infants [2, 3]. With the development and significant achievements in diagnosis and treatment in recent years, survival rates of preterm infants have been well improved; however, mortality rates of NEC remain largely unchanged [4, 5]. NEC is a multifactorial disease, with risk factors involving prematurity, formula feeding, bacterial colonization, and intestinal hypoxia/ischemia [6–8]. Single or combined factors as mentioned above trigger an inflammatory cascade, including the release of inflammatory mediators, migration and activation of neutrophils, and production of reactive oxygen species (ROS) [9]. Oxidative stress, defined as an imbalance between the overproduced ROS and the inability of antioxidants in the body, is intimately related to the occurrence of NEC. One study has found that severe NEC displays higher total oxidant status and oxidative stress index, which correlates positively to the degree of NEC severity [10]. The development and maturation of the antioxidant defense system are the most obvious events in the late gestational period; consequently, compared with term infants and children, preterm infants are more susceptible to ROS and have higher morbidity rates of NEC [11]. Oxidative stress causes cellular DNA damage, oxidation of lipids and proteins, and subsequently induces intestinal epithelial damage [9, 12, 13]. Despite our understanding of risk factors and oxidative stress, the actual cell death pathways of the intestinal epithelium, a critical event for the occurrence of NEC, remain incompletely elucidated.

In general, intestinal epithelial cells (IECs) barely die; however, severe intestinal tissue injury is observed both in the intestinal epithelium of NEC patients and in the ileum of NEC animal models [14]. The improper execution of cell death in IECs is fundamental to erosion of intestinal epithelium, which is the characteristic of inflammatory bowel disease and infectious colitis [15]. To explore the cell death pathways of intestinal injury during NEC, recent studies from our lab and others have shown that necroptosis is activated in the intestinal epithelium during NEC and participates in the inflammatory response and intestinal tissue injury [16, 17]. However, pharmacological inhibition of necroptosis fails to fully reverse NEC-initiated impairment in intestinal permeability and histopathological alterations [18], highlighting that there are other cell death pathways involved in NEC-induced intestinal injury. Parthanatos, like necroptosis, is a programmed inflammatory cell death and has been implicated in participating in ischemia/reperfusion-induced intestinal tissue injury [19, 20]. Poly (ADP-ribose) polymerase 1 (PARP1) dependence is one of the key features of parthanatos [21, 22]. PARP1 is the best-studied PARP enzyme, which is regarded as the DNA damage sensor via recognition of single-stranded and double-stranded DNA damage. On the one hand, in response to mild DNA damage PARP1 catalyzes nicotinamide adenine dinucleotide (NAD^+^) and adenosine triphosphate (ATP) to add ADP-ribose polymers to various nuclear proteins, assembling DNA repair proteins to initiate the DNA repair process [23, 24]; on the other hand, excessive DNA damage stimulates PARP1 hyperactivation, thereby increasing NAD^+^ and ATP consumption to form poly (ADP-ribose) (PAR). Subsequently, PAR translocates from the nucleus into the cytoplasm and mitochondria, leading to mitochondrial depolarization, and mitochondrial release and nuclear translocation of apoptosis-inducing factor (AIF), eventually causing chromatin condensation, DNA fragmentation, and cell death, as referred to parthanatos [25, 26].

Currently, the relationship between parthanatos and intestinal epithelial damage during NEC remains elusive. The present study aimed to ascertain whether PARP1-dependent parthanatos participates in intestinal epithelial damage during NEC and the underlying mechanism(s) involved by combined assessments and analyses of samples collected from human infants with NEC, animal blood and intestinal tissues collected from an established in vivo murine NEC model, and cellular specimens collected from an in vitro model of NEC.

## Results

### The presence of parthanatos in the intestinal tissues of human infants with NEC

**Fig. 1:**
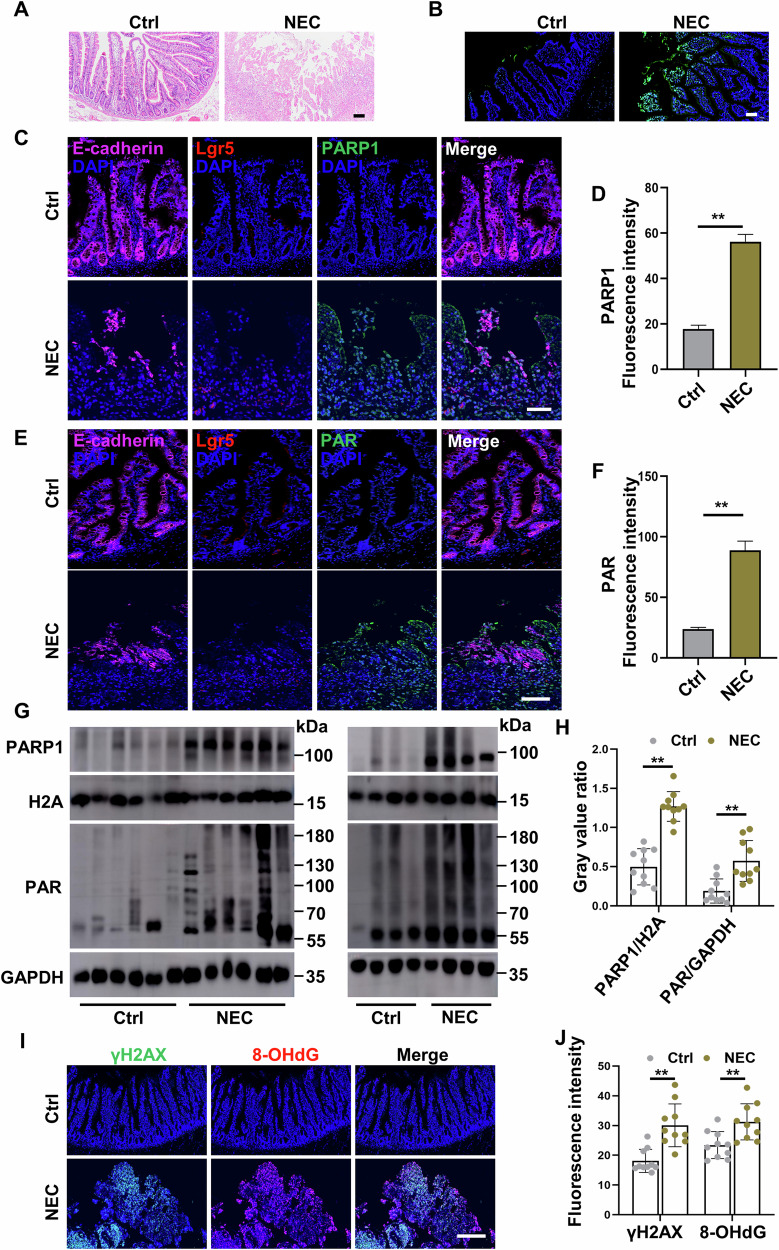
The presence of parthanatos in the intestinal tissues of human infants with NEC. Intestinal injury and expression of parthanatos-associated proteins were evaluated in human small intestines collected from infants with NEC and infants with congenital intestinal atresia as the control subjects. **A** Histology (H&E staining) of intestine tissues of congenital intestinal malformation and NEC. Scale bar: 100 μm. **B** TUNEL staining of intestine tissues of congenital intestinal malformation and NEC. Scale bar: 50 μm. **C**–**F** Expression of E-cadherin (magenta), Lgr5 (red), PARP1 (green) and PAR (green) in intestinal tissues between NEC patients and control subjects. Scale bar: 50 μm. **G**, **H** Western blot analysis and quantification of PARP1 and PAR expression in intestinal tissues between NEC patients and control subjects (*n* = 10 per group). **I**, **J** Immunofluorescent staining and quantification for DNA damage markers, γH2AX (green) and 8-OHdG (red), in intestinal tissues between NEC patients and control subjects (*n* = 10 per group). Scale bar: 50 μm. Western blots were repeated 3 times. Data are expressed as mean ± SD (^**^p < 0.01). Ctrl, control; DAPI, 4,6-diamidino-2-phenylindole.

To ascertain whether parthanatos is activated in human infants with NEC, intestinal tissue samples were collected from infants with NEC or infants with congenital intestinal atresia as the control subjects, and subjected to H&E, TUNEL, immunofluorescent staining, and western blot analysis. Firstly, profound morphological alterations in the intestinal structure with markedly increased epithelial cell death were observed in infants with NEC compared to the control subjects (Fig. 1A, B). Additionally, immunofluorescent staining results revealed an increased expression of parthanatos-associated proteins PARP1 and PAR at both the top and bottom of the villi, as identified by the Lgr5 marker, an epithelial stem cell marker, in NEC intestinal tissues (*p* < 0.01 versus control subjects) (Fig. 1C–F). Intestinal sections from infants with NEC also exhibited disrupted expression and localization of E-cadherin, an intestinal epithelial marker (Fig. 1C, E). Western blot analysis found substantially elevated expressing levels of both PARP1 and PAR proteins in intestinal tissues collected from infants with NEC in comparison with those collected from the control subjects (Fig. 1G, H). Furthermore, 8-OHdG and γH2AX, two DNA damage markers, were strongly overexpressed in the intestinal epithelium of infants with NEC compared to the control subjects (Fig. 1I, J). These data indicate that PARP1-dependent parthanatos is activated in the intestinal epithelium of NEC infants.

**Fig. 2:**
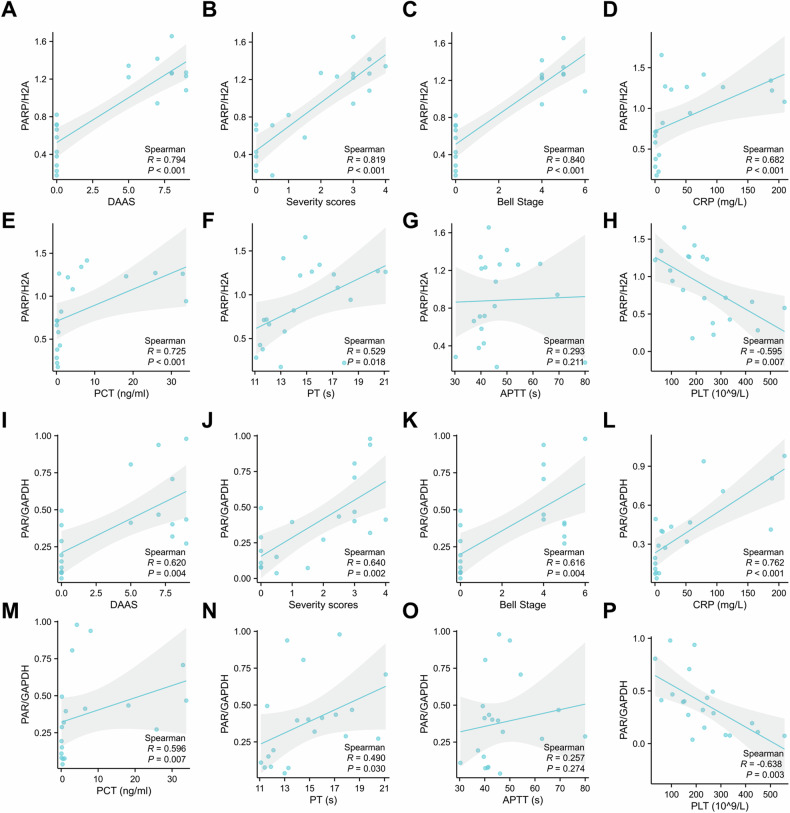
Upregulated PARP1 and PAR expression in NEC intestinal tissues correlates with clinical indices indicative of NEC incidence or severity. Correlations of the expression levels of PARP1 and PAR proteins in intestinal tissues collected from infants with NEC and infants with congenital intestinal atresia (*n* = 10 per group) with clinical indices including DAAS (**A**, **I**), intestinal severity scores (**B**, **J**), Bell stage (**C**, **K**), CRP (**D**, **L**), PCT (**E**, **M**), PT (**F**, **N**), APTT (**G**, **O**), and PLT (**H**, **P**) by Spearman’s correlation analysis. DAAS, the Duke abdominal assessment scale; Bell stage (I A = 1, I B = 2, II A = 3, II B = 4, III A = 5, and III B = 6); CRP C-reactive protein, PCT procalcitonin, PT prothrombin time, APTT activated partial thromboplastin time, PLT platelet.

We also established the correlations of PARP1 and PAR expression (Fig.1G, H) of 10 NEC patients and 10 patients with congenital intestinal atresia with the clinical indices (Supplementary Table 1). The Duke abdominal assessment scale (DAAS), an auxiliary index for NEC diagnosis, correlated positively with the expression levels of PARP1 (R = 0.794, *p* < 0.001) and PAR (R = 0.620, *p* = 0.004) (Fig. 2A, I). Similarly, intestinal histopathological severity scores, which were markedly elevated in NEC intestinal tissues, correlated positively with the expression levels of PARP1 (R = 0.819, *p* < 0.001) and PAR (R = 0.640, *p* = 0.002) (Fig. 2B, J). In addition, levels of protein expression in PARP1 (R = 0.840, *p* < 0.0010) and PAR (R = 0.616, *p* = 0.004) had significantly positive correlations with Bell stage (Fig. 2C, K). Moreover, serum levels of C-reactive protein (CRP) and procalcitonin (PCT), which were strongly elevated in NEC infants, also showed positive correlations with the expression levels of PARP1 (CRP: R = 0.682, *p* < 0.001; PCT: R = 0.762, *p* < 0.001) and PAR (CRP: R = 0.725, *p* < 0.001; PCT: R = 0.596, *p* = 0.007) (Fig. 2D, E, L, M). Coagulation-related indicator prothrombin time (PT) displayed modest positive correlations with the expression levels of PARP1 (R = 0.529, *p* < 0.018) and PAR (R = 0.490, *p* = 0.030) (Fig. 2F, N), whereas activated partial thromboplastin time (APTT) showed no significant correlations with PARP1 (R = 0.293, *p* = 0.211) and PAR (R = 0.257, *p* = 0.274) expression (Fig. 2G, O). In contrast, the number of platelets exhibited significant negative correlations with the expression levels of PARP1 (R= −0.595, *p* = 0.007) and PAR (R= −0.638, *p* = 0.003) (Fig. 2H, P). Collectively, these findings highlight the correlations between upregulated expression of PARP1 and PAR in NEC intestinal tissues and clinical indices indicative of NEC incidence or severity.

### PARP1-dependent parthanatos exists in the intestinal epithelium during experimental NEC

**Fig. 3:**
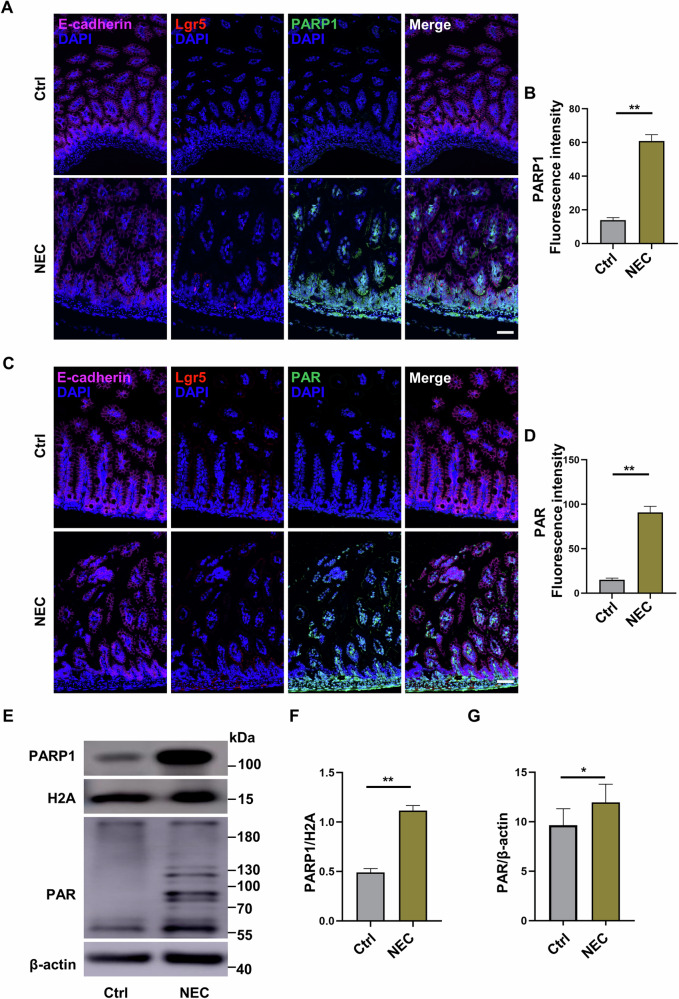
PARP1-dependent parthanatos exists in the intestinal epithelium during experimental NEC. C57BL/6 mice were exposed to hypoxia, formula gavage, and enteric bacteria for 96 hours, and the intestinal tissues were harvested. **A**–**D** Expression of E-cadherin (magenta), Lgr5 (red), PARP1 (green), and PAR (green) in intestinal ileum collected from control and NEC mice; nuclei were counterstained with DAPI. Scale bar: 50 μm. **E**–**G** Western blot analysis and protein quantification of PARP1 and PAR expression between control and NEC mouse ileum. Western blots were repeated 3 times. Data are expressed as mean ± SD (^*^p < 0.05 and ^**^p < 0.01). Ctrl control, DAPI 4,6-diamidino-2-phenylindole.

To investigate the existence of parthanatos in the intestinal epithelium and its potential role during NEC, we next employed an established murine model of NEC to analyze the expression of parthanatos-associated proteins PARP1 and PAR in intestinal tissues collected from the NEC mice and control mice. Consistent with the upregulated expression of PARP1 and PAR observed in the intestinal tissues of NEC infants, immunofluorescent staining showed that both PARP1 and PAR expression were substantially increased in the intestinal tissues of the NEC mice, compared to the control mice (Fig. 3A–D). We further used two protein markers, namely Lgr5, an epithelial stem cell marker, and E-cadherin, an epithelial cell marker, and found that the upregulated expression of PARP1 and PAR was predominantly distributed at the top and base of the villi, as marked by Lgr5, in intestinal tissues of NEC mice; meanwhile, the structure of intestinal villi was damaged and destroyed, as evidenced by the impaired expression and localization of E-cadherin in NEC mice (Fig. 3A–D). Western blot analysis further confirmed significantly increased protein expression of PARP1 and PAR in the intestinal tissues after induction of NEC (Fig. 3E–G), consistent with the findings seen in NEC infants (Fig. 1G, H). These results indicate that PARP1-dependent parthanatos exists in the intestinal epithelium during experimental NEC.

### Inhibition of parthanatos in vivo ameliorates NEC-initiated intestinal barrier injury and inflammation

**Fig. 4:**
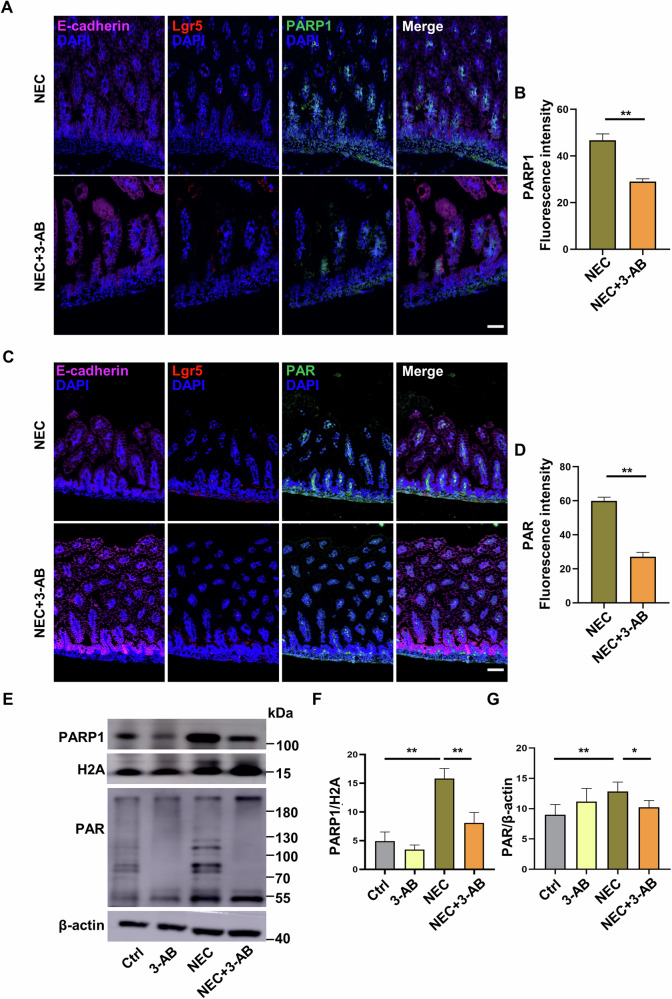
Administration of 3-AB inhibits parthanatos of the intestinal epithelium in NEC mice. Mice were injected intraperitoneally with 3-AB (20 μg/g body weight) for four consecutive days. **A**–**D** E-cadherin (magenta), Lgr5 (red), PARP1 (green), and PAR (green) in intestinal epithelial cells between NEC group and NEC + 3-AB group. Scale bar: 50μm. **E**–**G** Western blot analysis and protein quantification of PARP1 and PAR expression of intestine tissues. Western blots were repeated 3 times. Data are expressed as mean ± SD (^*^p < 0.05 and ^**^p < 0.01). Ctrl control, 3-AB 3-aminobenzamide, DAPI 4,6-diamidino-2-phenylindole.

We next examined whether parthanatos participates in intestinal epithelial damage during experimental NEC and furthermore, whether attenuation of parthanatos protects against NEC-associated intestinal barrier injury and inflammation. To this end, mice received an intraperitoneal injection of 3-AB, a PARP1 inhibitor. Upregulated PARP1 and PAR in the intestinal tissues were observed in NEC mice as revealed by immunofluorescent staining, whereas administration of 3-AB strongly attenuated NEC-enhanced PARP1 and PAR expression at the top and bottom of the intestinal villi, as identified by Lgr5 (*p* < 0.05, *p* < 0.01 versus NEC mice) (Fig. 4A–D). Western blot analysis showed that upon treatment with 3-AB, the increased expression of parthanatos-associated proteins PARP1 and PAR in NEC intestinal tissues was substantially attenuated (*p* < 0.05, *p* < 0.01 versus NEC mice) (Fig. 4E–G), demonstrating that administration of 3-AB suppresses the occurrence of parthanatos in the intestinal epithelium during experimental NEC.

**Fig. 5:**
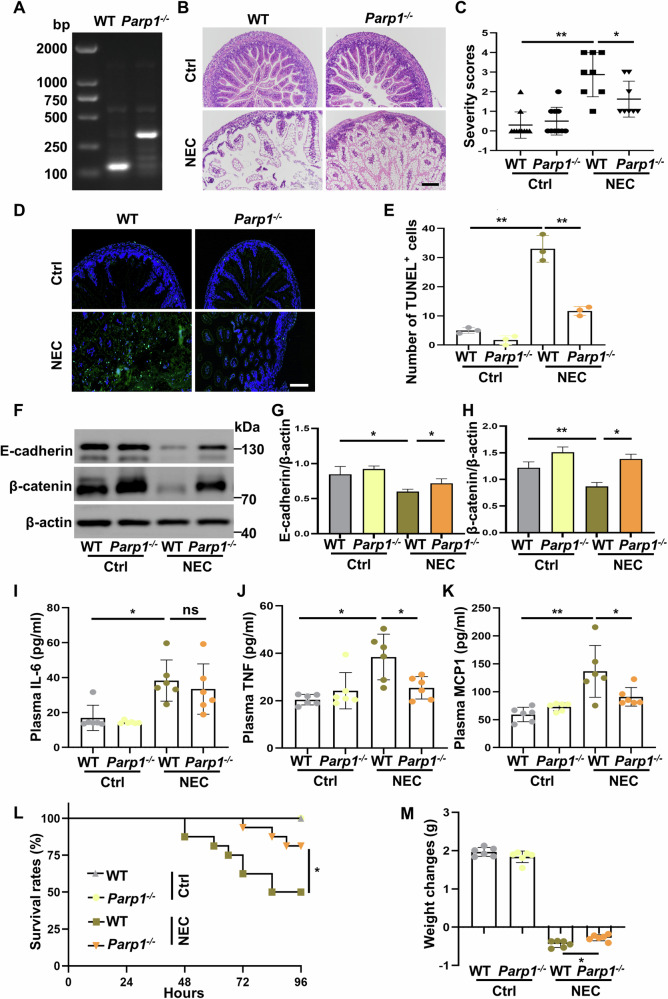
Inhibition of parthanatos by genetic knockout of *Parp1* in vivo protects against NEC-initiated intestinal barrier injury and inflammation. Blood and ileal samples were harvested 96 h after NEC induction. **A** Identification of *Parp1* knockout mice. **B** Representative images of H&E-stained intestinal sections. **C** Comparisons of histopathological severity scores in mouse ileum. **D**, **E** Representative images of TUNEL stained intestinal sections and number of TUNEL^+^ cells. Scale bar: 100 μm. **F**–**H** Expression and quantitative evaluation of β-catenin and E-cadherin in mouse ileum. **I**–**K** Plasma concentrations of IL-6, TNF-α, and MCP1 were assessed by a Cytometric Bead Array Mouse Inflammation kit (*n* = 6). **L** The Kaplan–Meier survival curve (*n* = 16 per group). **M** Weight changes of surviving pups in each group (*n* = 6). Western blots were repeated 3 times. Data are expressed as mean ± SD ^(ns^*p* > 0.05, ^*^p < 0.05 and ^**^p < 0.01). Ctrl control, WT wild-type.

Having demonstrated that parthanatos is activated in the intestinal epithelium during experimental NEC, we next attempt to clarify its potential role in NEC-initiated intestinal barrier injury and systemic inflammation. To assess parthanatos-associated intestinal epithelial damage during experimental NEC and the protection afforded by inhibition of parthanatos, we conducted experiments using *Parp1* knockout mice (Fig. 5A). Upon NEC modeling, the intestinal epithelial tissues in wild-type mice displayed prominent damage including disordered intestinal villus arrangement, villus breakage and exfoliation, and varying degrees of separation of the intestinal submucosa and lamina propria (Fig. 5B, C). TUNEL staining further showed markedly increased cell death of intestinal epitheliums in wild-type mice subjected to NEC (Fig. 5D, E). Of note, compared to the wild-type mice, NEC-induced intestinal epithelial damage in *Parp1*-deficient mice was substantially ameliorated, as evidenced by a relatively intact villus morphology without obvious disorder in intestinal villus arrangement and breakage, with significantly reduced intestinal histopathological severity scores (Fig. 5B, C) as well as markedly attenuated epithelial cell death (Fig. 5D, E). Epithelial barrier disruption is one of the key causes in the pathogenesis of NEC. We found that expression of E-cadherin and β-catenin, two proteins critical for maintaining intestinal barrier integrity and function, was markedly reduced in the ileum of wild-type mice subjected to NEC as revealed by western blot analysis, whereas *Parp1*-deficient mice partially reversed NEC-downregulated E-cadherin and β-catenin expression seen in wild-type mice (Fig. 5F–H).

Activation of inflammatory response is another important feature of NEC. Upon NEC modeling, plasma IL-6, TNF, and MCP1 levels were substantially elevated in wild-type mice; by contrast, *Parp1*-deficient mice displayed attenuated NEC-induced systemic inflammatory response with significantly reduced plasma TNF and MCP1, but not IL-6 (Fig. 5I–K). Critically, *Parp1*-deficient mice protected against NEC-associated lethality, with significantly improved survival from 50% in the wild-type mice to 81% (*p* < 0.05) (Fig. 5L). Moreover, *Parp1*-deficient mice showed ameliorated weight loss in response to NEC challenge compared to the wild-type mice (Fig. 5M). We observed a number of clinical features manifested during the induction of experimental NEC, including abdominal distention, emesis, and diarrhea, and notably, the severity of these symptoms was also mitigated in *Parp1*-deficient mice (data not shown). Collectively, these results indicate that inhibition of parthanatos alleviates intestinal epithelial damage and inflammatory reaction, thereby conferring protection in NEC mice.

### Induction of NEC in vitro in Caco-2 cells and human intestinal organoids induces parthanatos

**Fig. 6:**
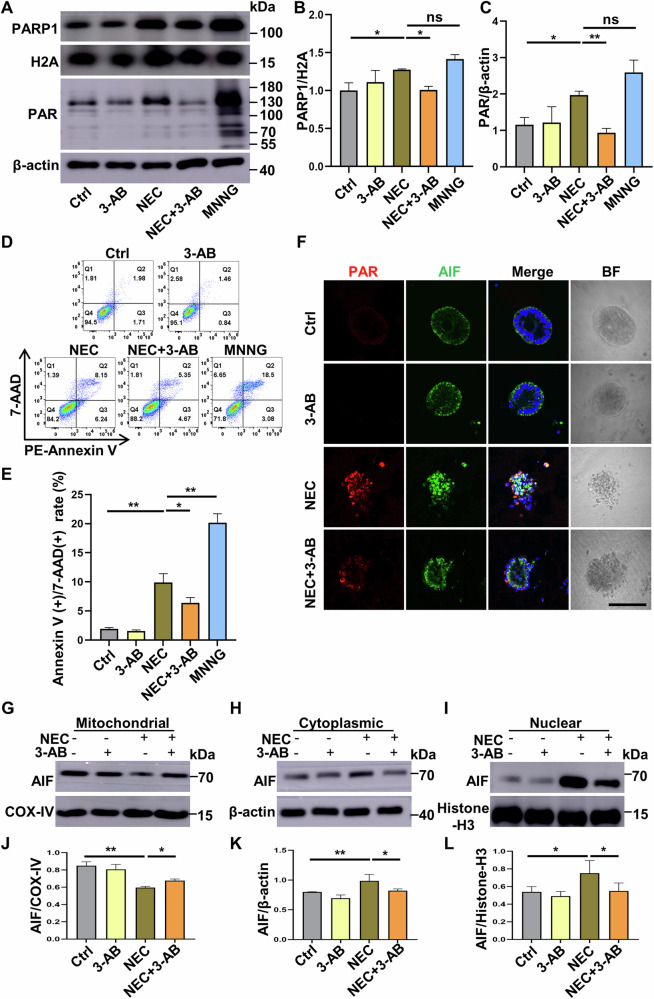
Induction of NEC in Caco-2 cells and human intestinal organoids leads to the occurrence of parthanatos. The induction of NEC with hypoxia and human enteric bacteria in vitro for 6 h (Caco-2) or 12 h (human intestinal organoids). **A**–**C** Western blot analysis and protein quantification of PARP1 and PAR expression in different groups; MNNG was used as a positive inducer of parthanatos. **D**, **E** FACScan analysis for detecting and quantifying cell death with double-positive staining of 7-ADD and Annexin V. **F** Confocal micrographs of control and NEC intestinal organoids pretreated with or without 3-AB (20 mM); fluorescent staining for PAR (red) and AIF (green) was shown. Scale bar: 100 μm. **G**–**L** Western blot analysis for distribution of AIF in subcellular fractions after NEC induction with or without 3-AB pretreatment. Western blots were repeated 3 times. Data are expressed as mean ± SD (^ns^*p* > 0.05, ^*^p < 0.05 and ^**^p < 0.01). Ctrl, control; 3-AB, 3-aminobenzamide; MNNG N-methyl-N′-nitro-N-nitrosoguanidine, BF bright field.

We utilized an in vitro NEC model where Caco-2 cells or human intestinal organoids were challenged with hypoxia plus human enteric bacteria for 6 h or 12 h to assess the presence of parthanatos and the protective effect of 3-AB. Induction of NEC in Caco-2 cells led to substantially enhanced expression of both PARP1 and PAR compared to the control Caco-2 cells (Fig. 6A–C), together with significantly increased cell death in Caco-2 cells characterized by double-positive staining for 7-ADD and Annexin V as revealed by FACScan analysis (Fig. 6D, E). Importantly, treatment of Caco-2 cells with MNNG, a positive inducer of parthanatos, resulted in upregulated PARP1 and PAR expression and massive cell death, as seen in Caco-2 cells subjected to in vitro NEC induction (Fig. 6A–E). Critically, treatment with 3-AB strongly downregulated PARP1 and PAR expression and attenuated cell death in Caco-2 cells challenged with hypoxia plus human enteric bacteria (Fig. 6A–E).

PARP1 functions as a DNA damage sensor, and upon excessive DNA damage, nuclear PARP1 promotes the formation of PAR. In turn, PAR translocates from the nucleus into the cytoplasm and mitochondria and binds to AIF in the mitochondria, thereby promoting AIF translocation into the cytoplasm and nucleus, a crucial step for the occurrence of parthanatos [27, 28]. Immunofluorescent staining confirmed the changes in the distribution of AIF in the mitochondria, cytoplasm, and nucleus. Specifically, the upregulated expression of PAR upon NEC induction entered the mitochondria and bound to AIF, thus facilitating AIF translocation into the nucleus and ultimately causing cell death in human NEC intestinal organoids and Caco-2 cells, which was partially alleviated by 3-AB intervention (Fig. 6F, Supplementary Fig. 1A). In addition, unlike the control and 3-AB-treated intestinal organoids, different degrees of nuclear condensation and dissolution were observed in both NEC intestinal organoids and NEC intestinal organoids treated with 3-AB (Fig. 6F).

Next, we isolated the mitochondrial, cytoplasmic, and nuclear components from Caco-2 cells and further quantified the expression and distribution of AIF in the mitochondrion, cytoplasm, and nucleus subcellular fractions by western blot analysis. As shown in Fig. 6G–L, induction of NEC in Caco-2 cells by means of incubation with hypoxia plus human enteric bacteria for 6 h substantially reduced AIF expression in the mitochondria, enhanced AIF expression in the cytoplasm and nucleus, and promoted AIF translocation from the mitochondria into the cytoplasm and nucleus, whereas treatment with 3-AB markedly inhibited the nuclear translocation of AIF in NEC Caco-2 cells. Additionally, the immunofluorescent staining results corroborated with the observed changes in the distribution of AIF within the mitochondria, cytoplasm, and nucleus (Supplementary Fig. 1A).

### ROS-induced DNA damage activates parthanatos in NEC-challenged Caco-2 cells and human intestinal organoids

**Fig. 7:**
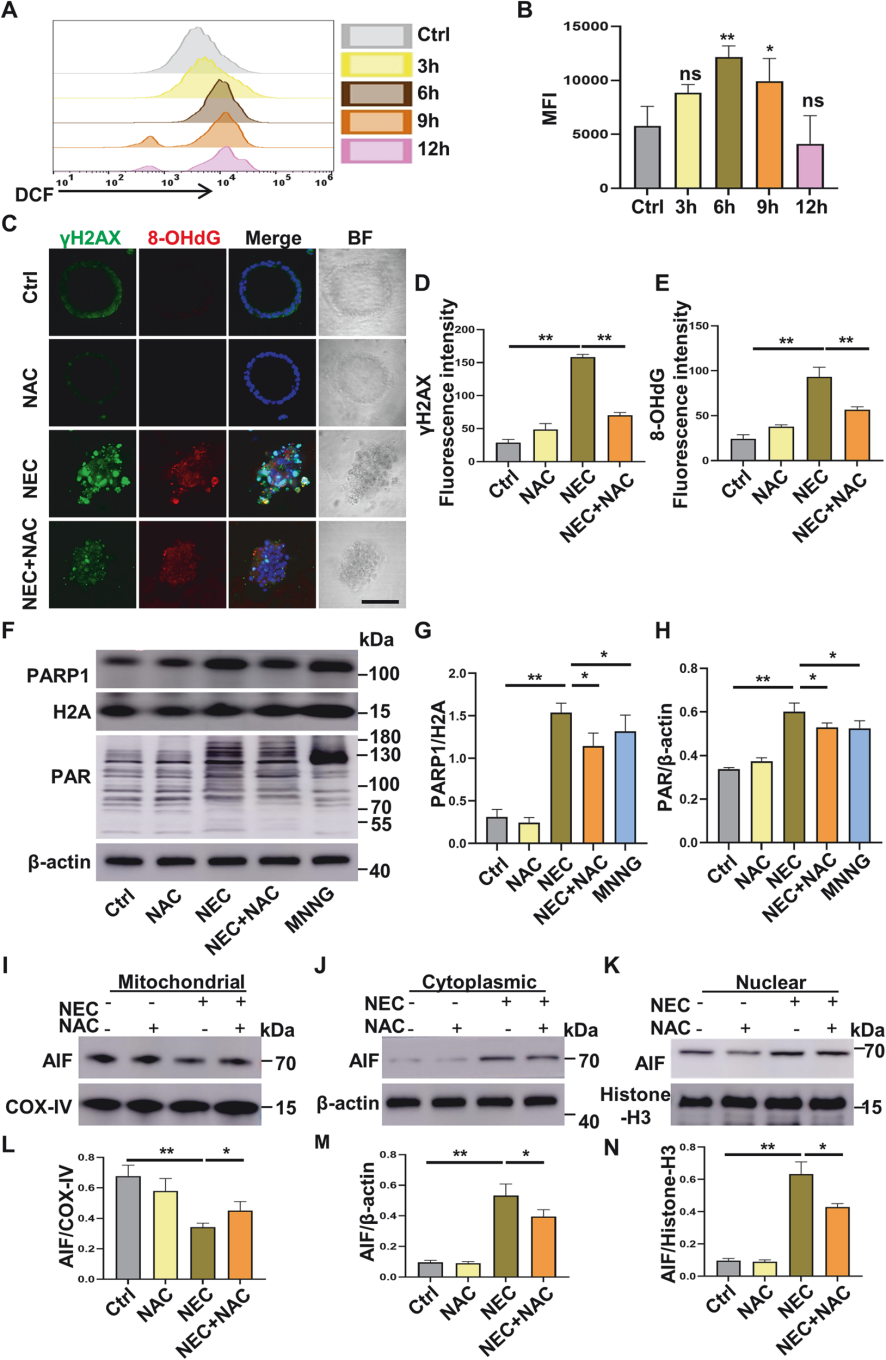
ROS-induced DNA damage activates parthanatos in NEC-challenged Caco-2 cells and human intestinal organoids. Human intestinal organoids and Caco-2 cells were stimulated with hypoxia and enteric bacteria for different time periods. **A**, **B** FACS analysis of DCFH-DA fluorescence to assess and quantify ROS generation for the indicated time points. **C** Confocal micrographs showing fluorescent staining of γH2AX (green) and 8-OHdG (red) in control and NEC intestinal organoids pretreated with or without NAC (5 mM). Scale bar: 100 μm. **D**, **E** Quantification of γH2AX and 8-OHdG fluorescent intensity in different groups. **F**–**H** Western blot analysis of PARP1 and PAR expression in different groups. **I**–**N** Western blot analysis for distribution of AIF in subcellular fractions after NEC induction with or without NAC pretreatment (5 mM). Western blots were repeated 3 times. Data are expressed as mean ± SD (^ns^*p* > 0.05, ^*^p < 0.05 and ^**^p < 0.01). Ctrl control, NAC N-Acetyl-l-cysteine, BF bright field.

To examine the involvement of ROS in DNA damage-activated parthanatos in an in vitro NEC model, we first challenged Caco-2 cells with hypoxia plus human enteric bacteria for different time points and measured intracellular ROS levels by FACScan analysis using a fluorescent probe DCF-DA. A time-dependent elevation in intracellular ROS with the peak level at 6 h was observed in the NEC-challenged Caco-2 cells compared to the control Caco-2 cells (*p* < 0.05, *p* < 0.01) (Fig. 7A, B). In addition, we monitored the dynamic changes of PARP1 and PAR protein expression within 12 h of NEC induction, and Western blot analysis demonstrated that following the induction of NEC, the expression levels of PARP1 and PAR proteins increased initially and then decreased, reaching the peak level at 6 h post NEC induction (Supplementary Fig. 2A–C). Increased intracellular ROS is known to induce DNA damage in different cell types and previous studies have shown that 8-OHdG and γH2AX are biomarkers of cellular DNA damage, and specifically, 8-OHdG is a marker associated with DNA oxidative damage [23, 29]. Isolated human intestinal organoids were pretreated with the antioxidant NAC (5 mM) for 1 h and further challenged with hypoxia plus human enteric bacteria for 12 h. As shown in Fig. 7C–E, both 8-OHdG and γH2AX were significantly increased in NEC-challenged intestinal organoids (*p* < 0.01 versus control intestinal organoids), whereas pretreatment with NAC substantially attenuated NEC-induced 8-OHdG and γH2AX expression (*p* < 0.01 versus NEC intestinal organoids). Critically, pretreatment of Caco-2 cells with NAC effectively downregulated NEC-enhanced expression in both PARP1 and PAR (Fig. 7F–H). Western blot analysis and immunofluorescent staining further revealed that pretreatment with NAC attenuated NEC-induced AIF release from the mitochondria and its translocation into the nucleus (Fig. 7I–N, Supplementary Fig. 1B). Taken together, these results demonstrate that ROS-induced DNA damage is involved in the occurrence of parthanatos in the intestinal epithelium during NEC.

## Discussion

**Fig. 8:**
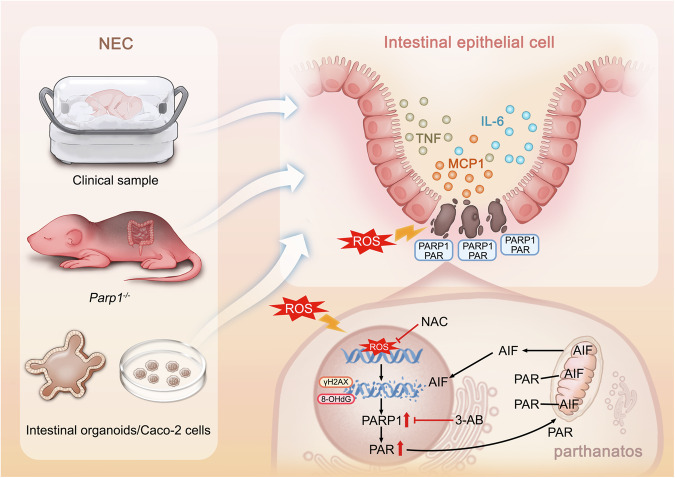
Proposed mechanism of parthanatos involved in the intestinal epithelial damage during NEC. The involvement of parthanatos in NEC-induced damage to intestinal epithelial cells was investigated by using clinical samples, human intestinal organoids, cell lines, and animal models. Both clinical and experimental findings from the present study demonstrate that the generation of ROS leads to severe DNA damage, which results in PARP1 accumulation and AIF translocation into the nucleus, consequently causing cell death such as parthanatos in the intestinal epithelium during NEC. Critically, genetically and pharmacologically inhibiting parthanatos or ROS, both in vivo and in vitro, demonstrates a mitigating effect on NEC-initiated intestinal epithelial damage and systemic inflammatory response.

NEC is a severe gastrointestinal emergency that predominantly affects preterm infants with extremely low birth weight, which is one of the main causes of neonatal death [30]. NEC, first described systematically by Arvo Ylppo in 1931, is characterized by an insidious clinical presentation, rapid progression, intestinal necrosis, and predisposition to peritonitis [31]. A considerable body of studies focusing on both experimental and clinical NEC has been carried out in recent decades, but the underlying mechanisms involved in the pathogenesis and progression of NEC are not fully understood. In the present study, we aimed to elucidate the role of parthanatos in NEC-initiated intestinal epithelial damage using mouse models, human intestinal cell line or intestinal organoids, and clinical samples. We show that ROS-induced DNA damage leads to strong PARP1 expression, thereby causing PAR formation, AIF nuclear translocation, and cell death in the intestinal epithelium of NEC. Critically, inhibition of parthanatos both in vivo and in vitro effectively alleviates NEC-associated intestinal epithelial damage and inflammatory reaction. A proposed mechanism of parthanatos involved in intestinal epithelial damage during NEC is illustrated in Fig. 8.

The intestine epithelium acts as the boundary between the inner body and the external environment. The abnormal pattern of and interactions between the intestinal microbiota play an important role in the initiation and pathogenesis of NEC [32, 33]. Several studies have found that the intestinal barrier function is impaired during NEC with increased intestinal permeability and downregulated expression of mucosal epithelial barrier proteins [34–36]. In the present study, we reported for the first time that parthanatos was activated and existed in the intestinal epithelium of both the NEC infants and experimental NEC in mice. Furthermore, *Parp1* knockout mice exhibited reduced damage in intestinal villi and restored intestinal barrier function, thereby protecting against NEC-associated lethality protection in mice with NEC. Li et al. found that receptor interacting protein kinase 1 (RIP1)-mediated necroptosis and PARP1-dependent parthanatos involve in ischemia/reperfusion-induced intestinal tissue injury, whereas combined inhibition of both RIP1 and PARP1 maximizes the improvement of intestinal epithelial damage [20]. Accumulated work has also indicated that inhibition of parthanatos protects against blood-brain barrier damage, edema, infarct size, and neuronal cell death through downregulation of oxidative stress and upregulation of antioxidant capacity [24, 37–39]. Therefore, inhibition of parthanatos could be considered as a potential therapeutic strategy for protecting tissue and organ injury in various pathological conditions.

In addition to apparent intestinal epithelial damage, there is an ongoing inflammatory response during NEC. It has been found that in both clinical NEC infants and animal NEC models plasma levels of proinflammatory cytokines are elevated, whereas anti-inflammatory cytokines are decreased [40–42]. In the present study we also observed substantially elevated plasma IL-6, TNF, and MCP1 in NEC mice, and these enhanced proinflammatory cytokines and chemokine were effectively attenuated by blocking parthanatos genetically. Several studies have shown that oxidative stress in ischemia/reperfusion generates massive ROS, leading to the development of NEC [43–45]. In the present study, we found that intracellular ROS initially increased and reached its peak level at 6 h in Caco-2 cells challenged with hypoxia and enteric bacteria. Importantly, we observed that 8-OHdG and γH2AX, two biomarkers of cellular DNA damage, were substantially increased in intestinal organoids following induction of NEC, whereas both 8-OHdG and γH2AX were downregulated by treatment with an antioxidant NAC. Sevoflurane exposure caused severe DNA damage, whereas inhibition of ROS reduced the occurrence of parthanatos in neuronal cells [29]. Similarly, our results also suggest that ROS-induced DNA damage might be an upstream mechanism of parthanatos during the development of NEC.

Intestinal tissue injury is a characteristic of NEC, which can lead to significant necrosis in severe cases. Accumulated evidence has indicated that apoptosis, pyroptosis, and necroptosis are involved in cell death of the intestinal epithelium [17, 46, 47]. In the present study, FACScan analysis showed an increasing number of dead cells over time, suggesting that intestinal epitheliums undergo severe cell death during NEC, while inhibition of parthanatos attenuates NEC-initiated cell death. PARP1 is regarded as a DNA damage sensor. Notably, our results demonstrate that ROS-induced DNA damage activates PARP1-dependant parthanatos, as represented by the formation of PAR, binding of PAR to AIF, and subsequent nuclear translocation of AIF, and critically, inhibition of PARP1 with 3-AB alleviates the occurrence of parthanatos by attenuating AIF translocation into the nucleus with ameliorated cell death. PAR can be degraded by PAR glycohydrolase, which leads to a decrease in AIF release and inhibition of MNNG-induced parthanatos, thereby improving middle cerebral artery occlusion [25, 48]. It has been revealed that the D3 domain of AIF contains a key binding site for PAR [21]. Thus, the binding of PAR to AIF is crucial during parthanatos; however, the exact binding mechanism is not fully understood.

Collectively, our results demonstrate for the first time that parthanatos plays a potential role in the pathogenesis of NEC. Data from both in vivo and in vitro models suggest that inhibition of parthanatos both pharmacologically and genetically may act as a key intervention in ameliorating intestinal epithelial damage and inflammatory responses during NEC. Our findings provide important insights into the molecular mechanism(s) of NEC and offer the possibility in preventing and treating NEC through inhibition of parthanatos.

## Materials and methods

### Reagents and antibodies

3-aminobenzamide (3-AB, M1785) and 1-Methyl-3-nitro-1-nitrosoguanidine (MNNG, M9797) were purchased from AbMole (Houston, TX, USA). N-Acetyl-l-cysteine (NAC, A7250) and lipopolysaccharide (LPS, L4524) were purchased from Sigma-Aldrich (Darmstadt, Germany). Human IntestiCult™ Organoid Growth Medium (06010) and Gentle Cell Dissociation Reagent (GCDR, 07174) were obtained from Stem Cell (Vancouver, Canada). Matrigel (356231) was from Corning (New York, NY, USA). Antibodies for PARP (9532), AIF (5318), β-catenin (9562), E-cadherin (3195), GAPDH (5174), and β-actin (4970) were obtained from Cell Signaling Technology (Boston, MA, USA). Anti-COX IV (ab202554), Occludin (ab216327), Lgr5 (ab273092), Histone H2A (ab18255) and Histone H3 (ab1791) were purchased from Abcam (Cambridge, UK). Anti-PAR (AM80) was obtained from Merck Millipore (Billerica, MA, USA). Anti-8-OHdG (sc66036) was from Santa Cruz Biotechnology (Dallas, TX, USA).

### Human intestinal tissue specimens

Samples of human intestines were obtained from informed and consenting patients undergoing abdominal surgery for NEC and intestinal atresia. All experimental protocols were approved by the Medical Ethics Committee at the Children’s Hospital of Soochow University (No. 2021CS091).

### Mice

All experiments performed in this study were reviewed and approved by the Ethical Committee of Soochow University (No. SUDA20230625A05) and were in line with the Guide for the Care and Use of Laboratory Animals published by the US National Institutes of Health (No. 85-23, revised in 1996). C57BL/6 mice were purchased from JOINN Laboratories (Suzhou, China, License No. SCXK (Su) 2018-0006) and housed in the specific-pathogen-free (SPF) environment. *Parp1* knockout mice were gifts from Institutes for Translational Medicine at Suzhou Medical College, Soochow University, Suzhou, Jiangsu. All mice were allocated to the groups randomly. After executing the mice, blood samples were collected through cardiac puncture and the terminal ileum was harvested by an abdominal incision for subsequent experiments. Genotypes of the mice were verified using the following primers: wild-type allele detection (112 bp): 5′-CATGTTCGATGGGAAAGTCCC-3′ and 5′- CCAGCGCAGCTCAGAGAAGCCA-3′; knockout allele detection (350 bp): 5′-CATGTTCGATGGGAAAGTCCC -3′ and 5′- AGGTGAGATGACAGGAGATC -3′.

### Human intestinal organoids

Human intestinal organoids from the ileum of congenital intestinal atresia patients with the non-inflammatory condition were isolated, cultured, passaged, and frozen according to established protocols [27, 49]. Briefly, isolated intestinal organoids were with PBS, minced, and digested in Gentle Cell Dissociation Reagent (GCDR). Following centrifugation and filtration, the Matrigel®-crypt suspension of intestinal organoids was plated in 24 well plates, cultured in the DMEM/F12 medium with a replacement every 2–4 days and passaged every 7–10 days.

### Caco-2 cell line

The human Caco-2 cell line was purchased from the Cell Bank of Type Culture Collection of the Chinese Academy of Sciences (Shanghai, China). Cell line was routinely tested for the presence of mycoplasma contamination. Cells were cultured in DMEM/F12 medium supplemented with 10% fetal bovine serum and 1% penicillin/streptomycin.

### NEC models

An in vivo NEC model was induced in 7-day-old C57BL/6 mice as previously described with some modifications [28, 50]. Briefly, NEC mice were fed with the formula (40 μl/g of body weight) consisting of Similac Advance infant formula (Abbott Nutrition, Columbus, OH, USA): Esbilac (PetAg) canine milk replacer in a ratio of 2: 1 via oral gavage three times a day. The formula was supplemented with enteric bacteria isolated from human infants with severe NEC (12.5 μl original stool slurry in 1 ml formula). The mice were subjected to 10 min of hypoxic stress in a hypoxia chamber containing 95% N_2_ and 5% O_2_ twice a day. Induction of experimental NEC in vivo by means of the formula feeding and enteric bacteria plus hypoxia challenges lasted 4 days. Daily intraperitoneal injection of 3-AB (20 μg/g of body weight) was carried out for the 3-AB and NEC + 3-AB groups.

An in vitro NEC model was induced in Caco-2 cells and intestinal organoids. Briefly, the cell culture medium for Caco-2 cells and intestinal organoids was replaced with the medium without antibiotics. Single enteric bacterial colonies isolated from the stool of an NEC infant were inoculated in DMEM/F12 medium and shaken at 37 °C overnight. Caco-2 cells and intestinal organoids were infected with 5 × 10^6^ CFU of single enteric bacterial colonies as previously described [51]. Infected Caco-2 cells and intestinal organoids were further challenged with hypoxia by using a hypoxic incubator (5% CO_2_ and 1% O_2_, 37 °C) for 6 h and 12 h, respectively [17]. In addition, 3-AB (20 mM) or NAC (5 mM) were added in 3-AB-treated or NAC-treated groups 1 h before infection and hypoxia.

### Immunofluorescence

Terminal ileal tissues were fixed in 4% paraformaldehyde at room temperature for 24 h, then they were subjected to dehydration, clearing, paraffin embedding procedures, and cut into 5 μm-thick sections. Sections were deparaffinized, gradient alcohol dehydrated, antigen repaired, and further rinsed three times at an interval of 5 min. Subsequently, paraffin sections were stained with various primary antibodies overnight at 4 °C and then stained with secondary antibodies. Nuclei were stained with DAPI, and the slides were observed under a fluorescence microscope (Olympus, New York, NY, USA).

Intestinal organoids were cultured in confocal dishes. After treatment, organoids were fixed with 4% paraformaldehyde for 20 min at room temperature, incubated in 0.5% Triton X-100 for 15 min, and blocked with 3% BSA in 0.3% Triton X-100 for 1 h. All other steps were the same as the paraffin sections as described above. Hochest33342 was used to stain the nuclei. Fluorescence was visualized with a confocal microscope (Olympus) and fluorescence intensity was analyzed using Image J software (NIH, Bethesda, MD, USA).

### Hematoxylin and eosin (H&E) staining

Paraffin sections (5 μm) were dehydrated and stained with hematoxylin and eosin to assess the degree of intestinal injury. Two blinded investigators assessed intestinal histological scores, and their average score was calculated. According to the scoring system, a definitive diagnosis of NEC was given if the grade of intestinal histological scores ≥ 2 [52, 53].

### Western blot analysis

Protein expression levels were measured by western blot analysis. Briefly, samples were lysed in RIPA lysis buffer (Beyotime Biotechnology, Shanghai, China), lysates were centrifugated, and the resultant supernatants were collected. Mitochondrial, cytosolic, and nuclear proteins were extracted using the protein extraction kits (Beyotime Biotechnology). Lysates were extracted by 7.5–10% SDS-PAGE and transferred onto a PVDF membrane (Millipore, Schwalbach, Germany). The membrane was blocked for 2 h at room temperature with 5% nonfat milk, incubated with the different primary antibodies overnight at 4 °C and secondary antibodies for 1 h at room temperature. Blots were visualized with an ECL reagent (Thermo Fisher, Waltham, MA, USA) and quantification of protein bands was performed with Image J software (NIH) [54].

### Plasma cytokines and chemokines measurement

Blood samples were harvested from mice by cardiac puncture and centrifugated to obtain supernatants. Serum levels of IL-6, TNF, and MCP1 were quantified using the Cytometric Bead Array (CBA) kit (BD Biosciences, Franklin Lakes, NJ, USA) according to the manufacturer’s instructions.

### Intracellular ROS measurement

Intracellular ROS levels were detected using the ROS-sensitive fluorescent dye, DCFH according to ROS Assay Kit (Beyotime Biotechnology). Briefly, cells were seeded in 6-well plates and exposed to hypoxia and NEC enteric bacteria for different time periods. Afterward, cells were collected using pancreatic enzyme and incubated with 1 μM DCFH-DA for 20 min at 37 °C. Flow cytometry (Immunotech Beckman Coulter, Brea, CA, USA) was performed to analyze the 488/FITC^+^ population and analyzed using Flow Jo software (Tree Star Inc, Wokingham, UK) [55].

### Cell death assessment by FASCcan analysis

7-Amino-Actinomycin (7-AAD) and annexin V staining were used for the assessment of cell death. After cells were treated at different time intervals, cells were stained with 5 µl Annexin V and 5 µl 7-AAD for 15 min at room temperature using a PE Annexin V Apoptosis Detection Kit I (BD Biosciences), and assessed by FACScan analysis within 1 h using Flow Jo software (Tree Star Inc) [56]. Dead cells were positive for both Annexin V and 7-AAD.

### Statistical analysis

All data are expressed as the mean ± SD. Statistical analysis was analyzed using GraphPad Prism 9.0.0. Statistical significance between two groups of data was determined using the unpaired two-tailed Student *t*-test. Multiple grouped data were evaluated using analysis of variance (ANOVA) with Tukey’s multiple comparison test. Survival was analyzed by the Kaplan–Meier and log rank test. For all results, *p* < 0.05 was considered significant.

### Supplementary information

Supplementary Figures

Original full length western blots

Supplementary Table 1

## Data Availability

All data that support the findings of this study are included in the article.
