# Supplementary material for: Parthanatos initiated by ROS-induced DNA damage is involved in intestinal epithelial injury during necrotizing enterocolitis
Source: Cell Death Discov. 2024 Jul 31;10:345. doi: 10.1038/s41420-024-02114-z (PMC11291915; doi:10.1038/s41420-024-02114-z)
Supplement: Supplementary file 1 — Supplementary Figures [file 41420_2024_2114_MOESM1_ESM.docx]

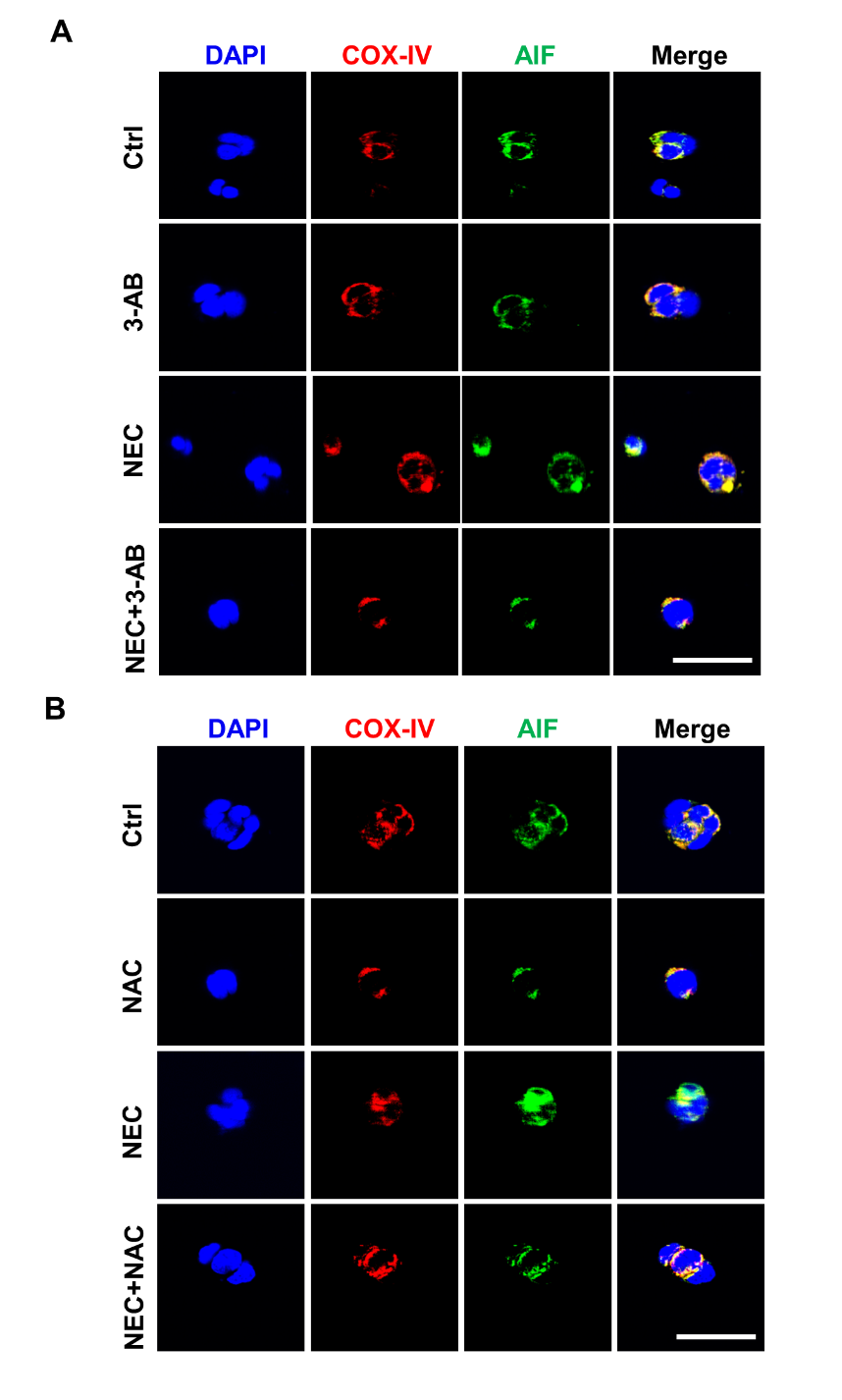


**Supplementary Figure 1. The distribution of AIF within the mitochondria, cytoplasm, and nucleus in Caco-2 cells.** Caco-2 cell**s** were exposed to hypoxia and human enteric bacteria for 6 h to induce *in vitro* NEC. (A) Confocal micrographs of control and NEC Caco-2 cells pretreated with or without 3-AB (20 mM); fluorescent staining for COX-IV (red) and AIF (green) was shown. Scale bar: 50μm. (B) Confocal micrographs of control and NEC Caco-2 cells pretreated with or without NAC (5mM); fluorescent staining for COX-IV (red) and AIF (green) was shown. Scale bar: 50μm. Ctrl, control; 3-AB, 3-aminobenzamide; NAC, N-Acetyl-l-cysteine.


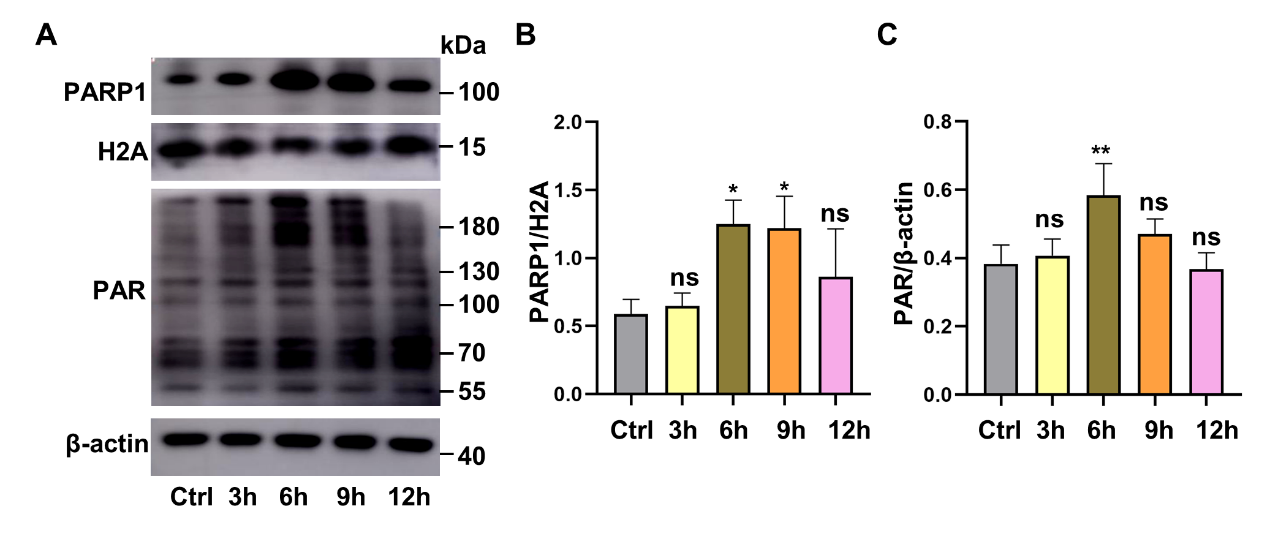


**Supplementary Figure 2. The expression of PARP1 and PAR proteins in Caco-2 cells over time upon in vitro NEC modeling.** Caco-2 cell**s** were challenged with hypoxia and human enteric bacteria to induce in vitro NEC. (A-C) Western blot analysis and quantification of PARP1 and PAR protein expression following NEC induction. Western blots were repeated 3 times. Data are expressed as mean ± SD (^ns^p>0.05, *p<0.05 and **p<0.01). Ctrl, control.
