# Supplementary material for: Parthanatos initiated by ROS-induced DNA damage is involved in intestinal epithelial injury during necrotizing enterocolitis
Source: Cell Death Discov. 2024 Jul 31;10:345. doi: 10.1038/s41420-024-02114-z (PMC11291915; doi:10.1038/s41420-024-02114-z)

Figure 1G-PARP1

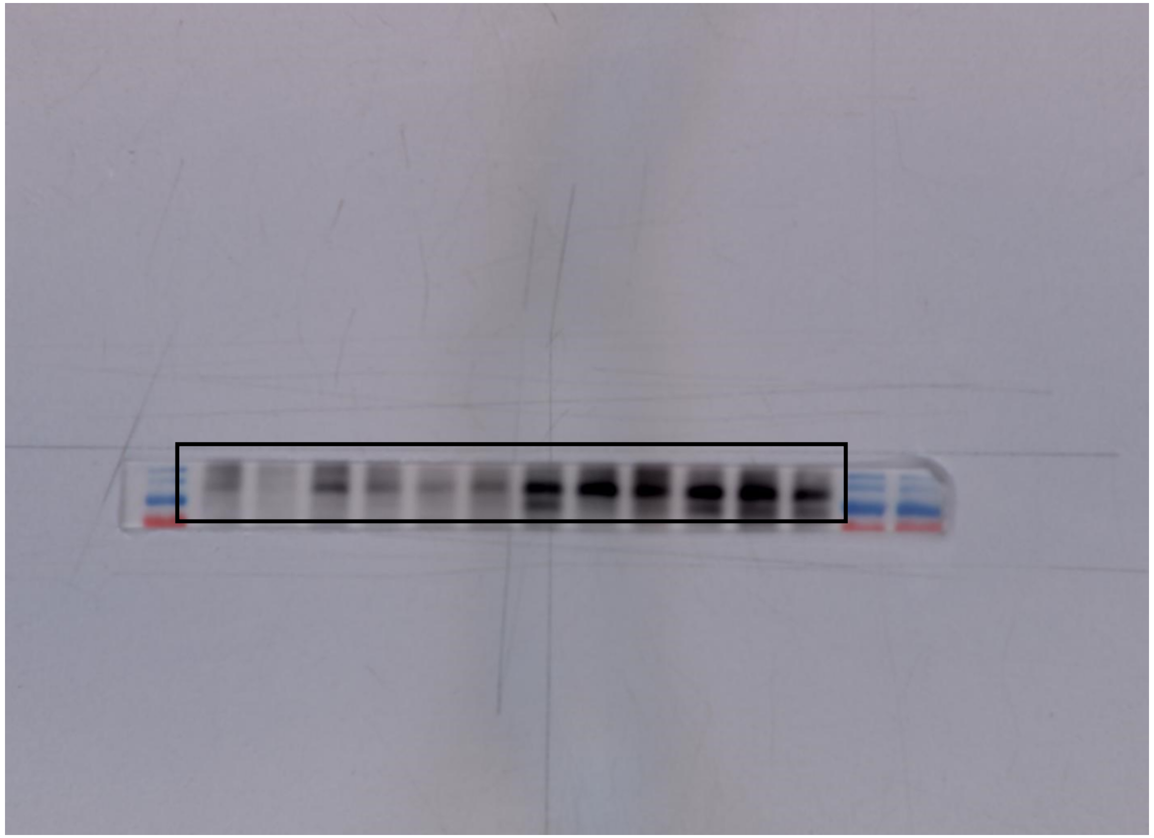

Figure 1G-H2A

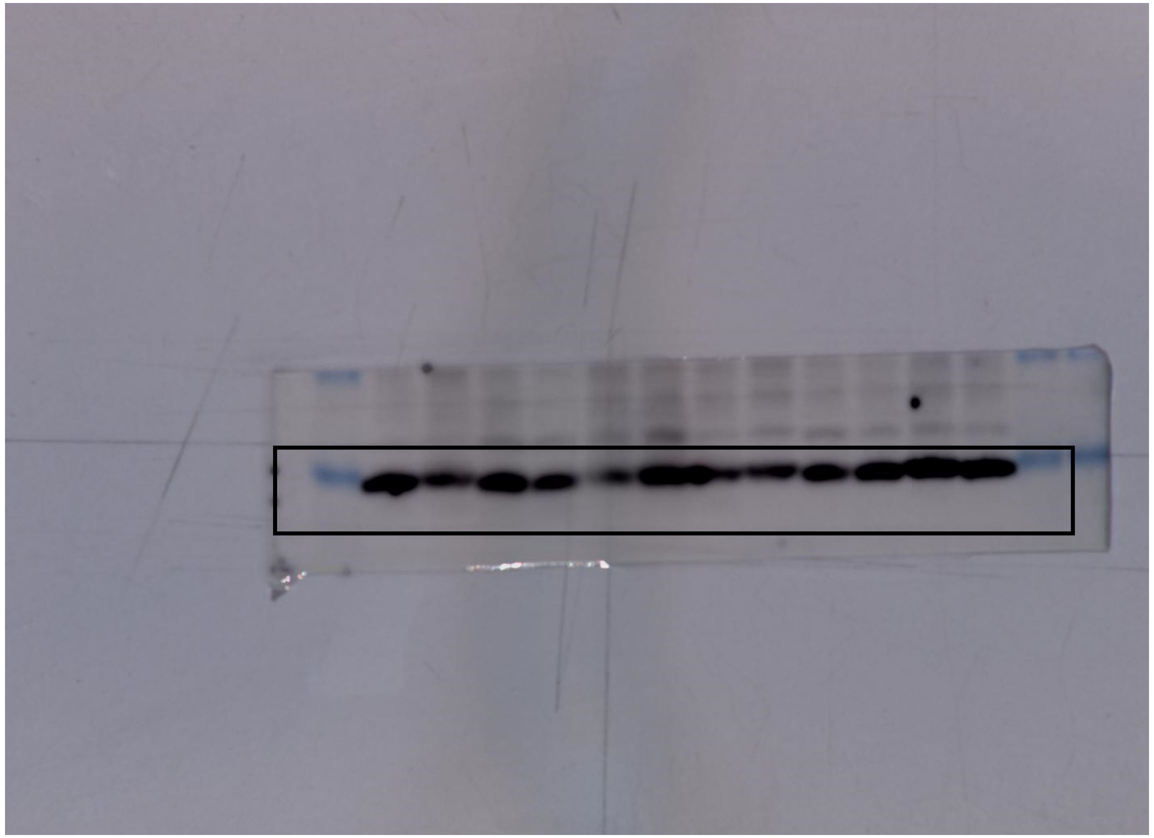

Figure 1G-PARP1

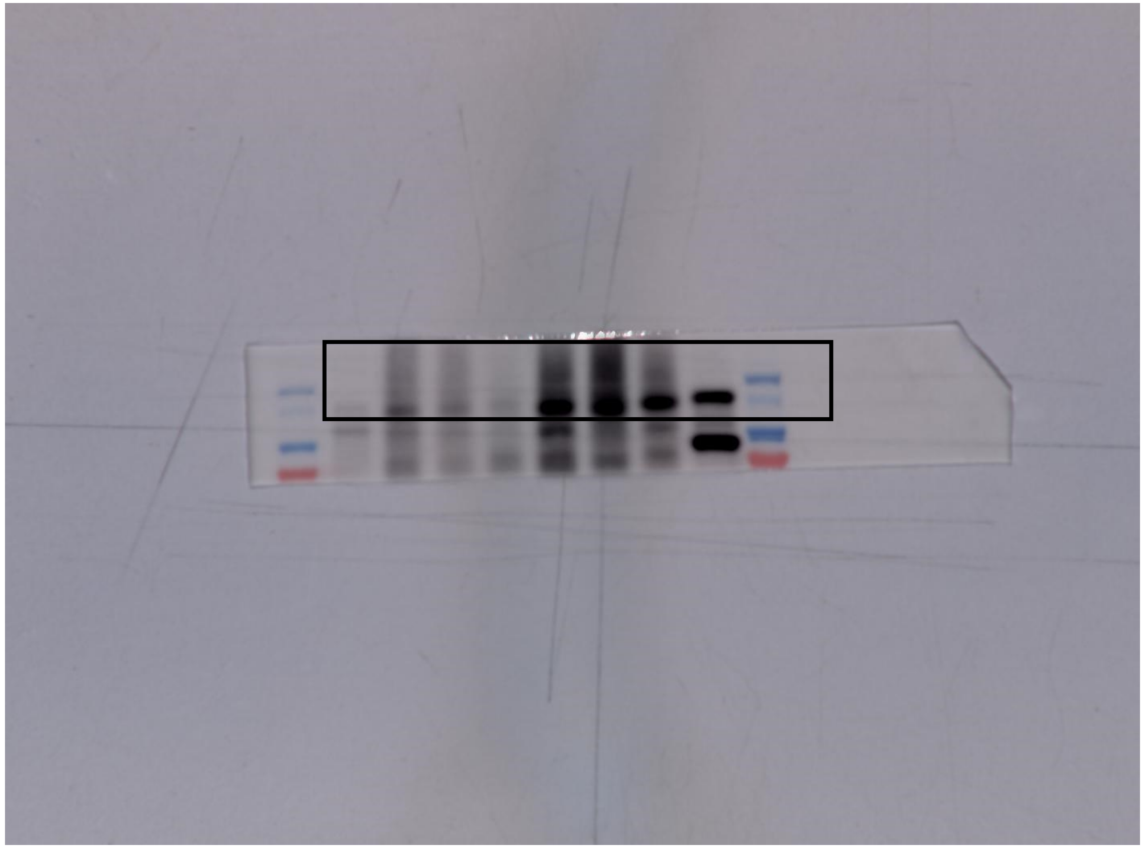

Figure 1G-H2A

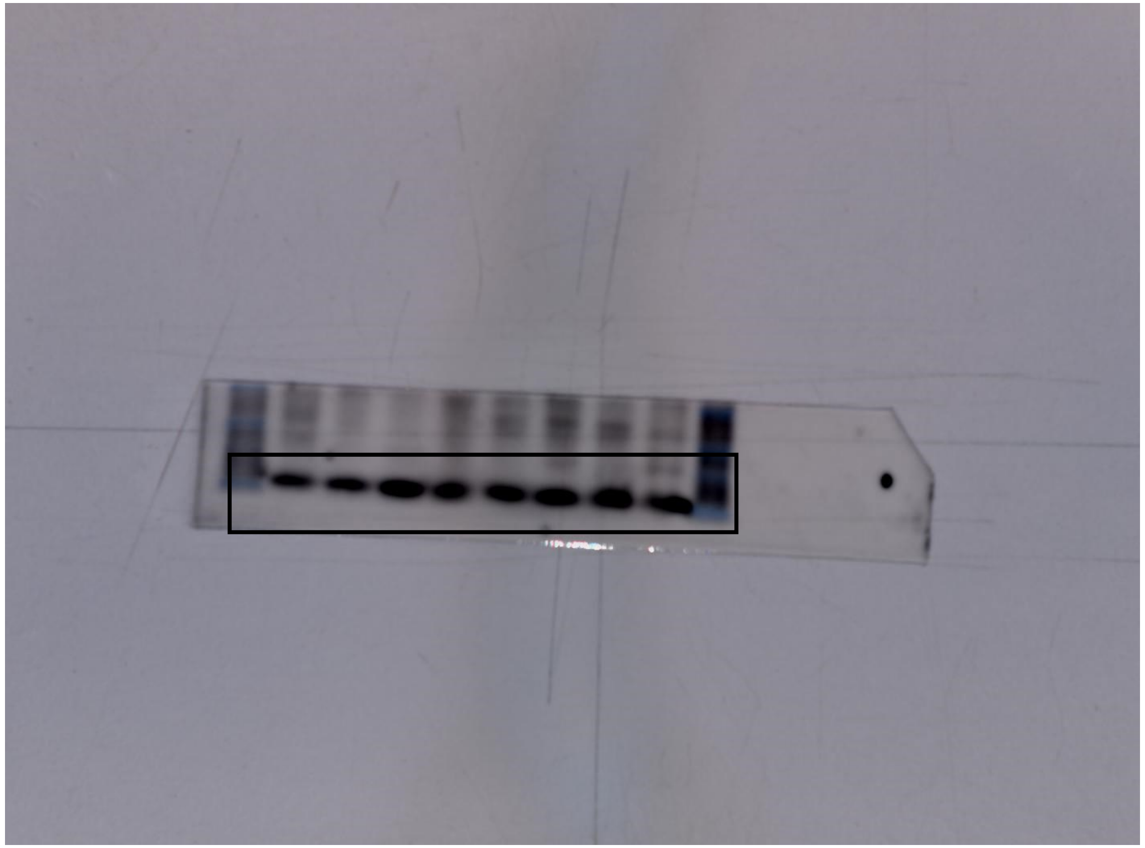

Figure 1G-PAR

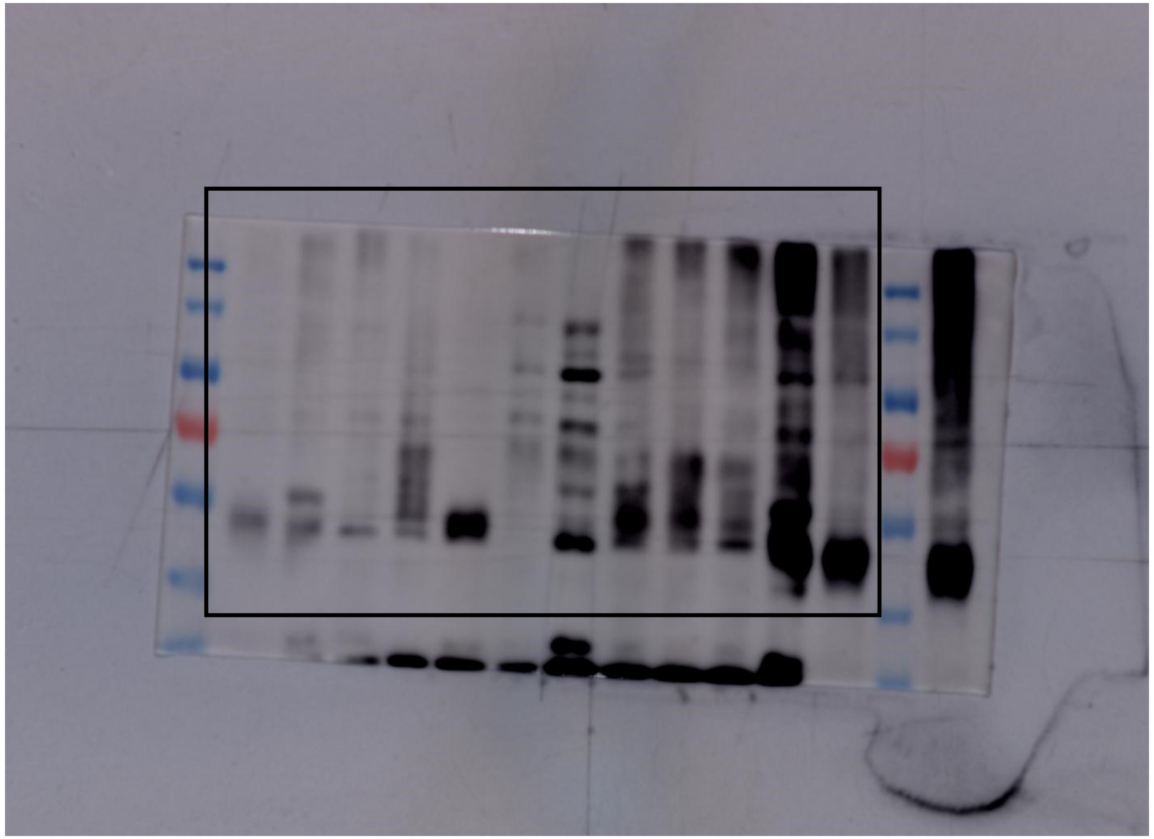

Figure 1G-GAPDH

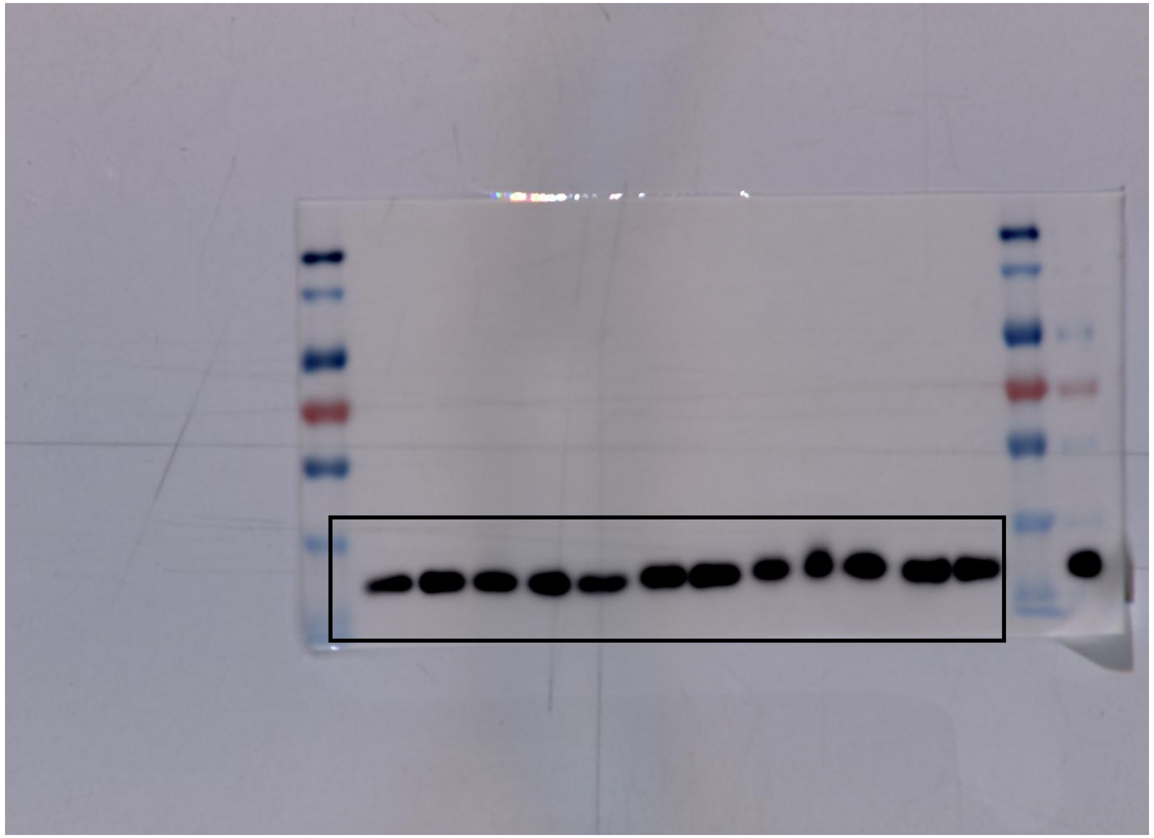

Figure 1G-PAR

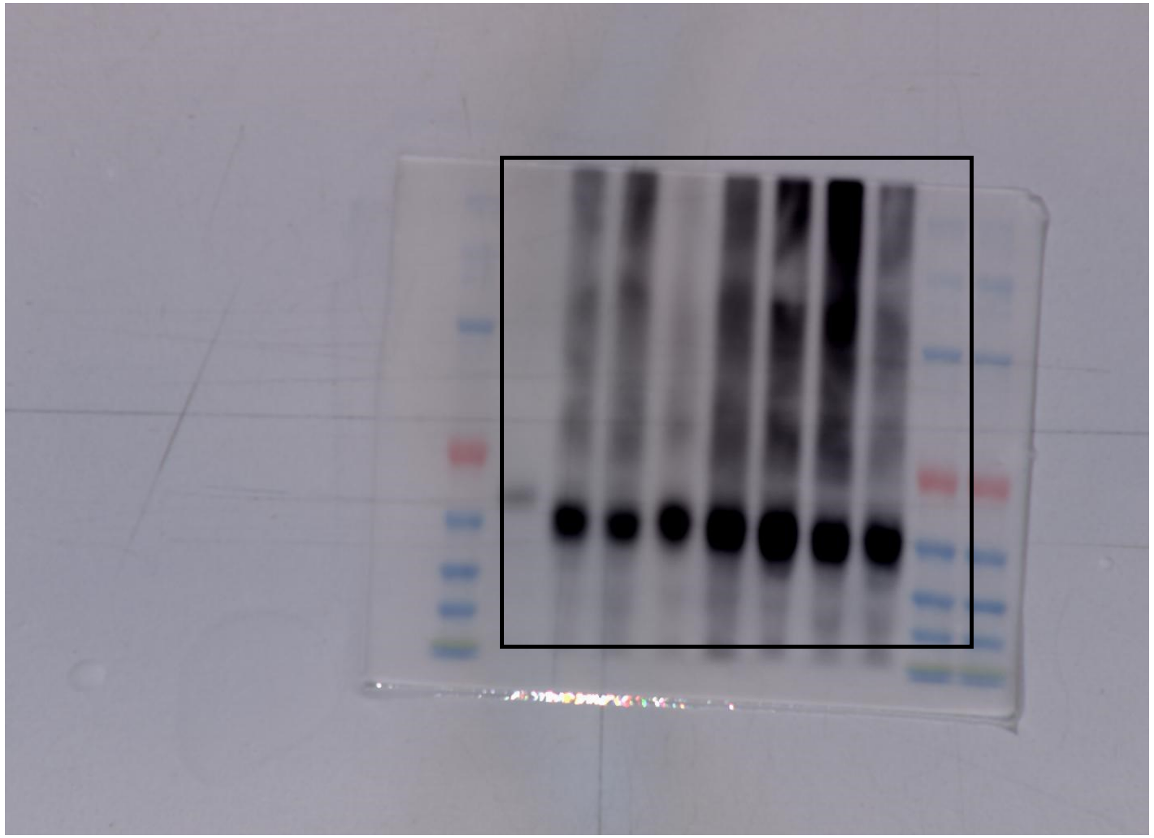

Figure 1G-GAPDH

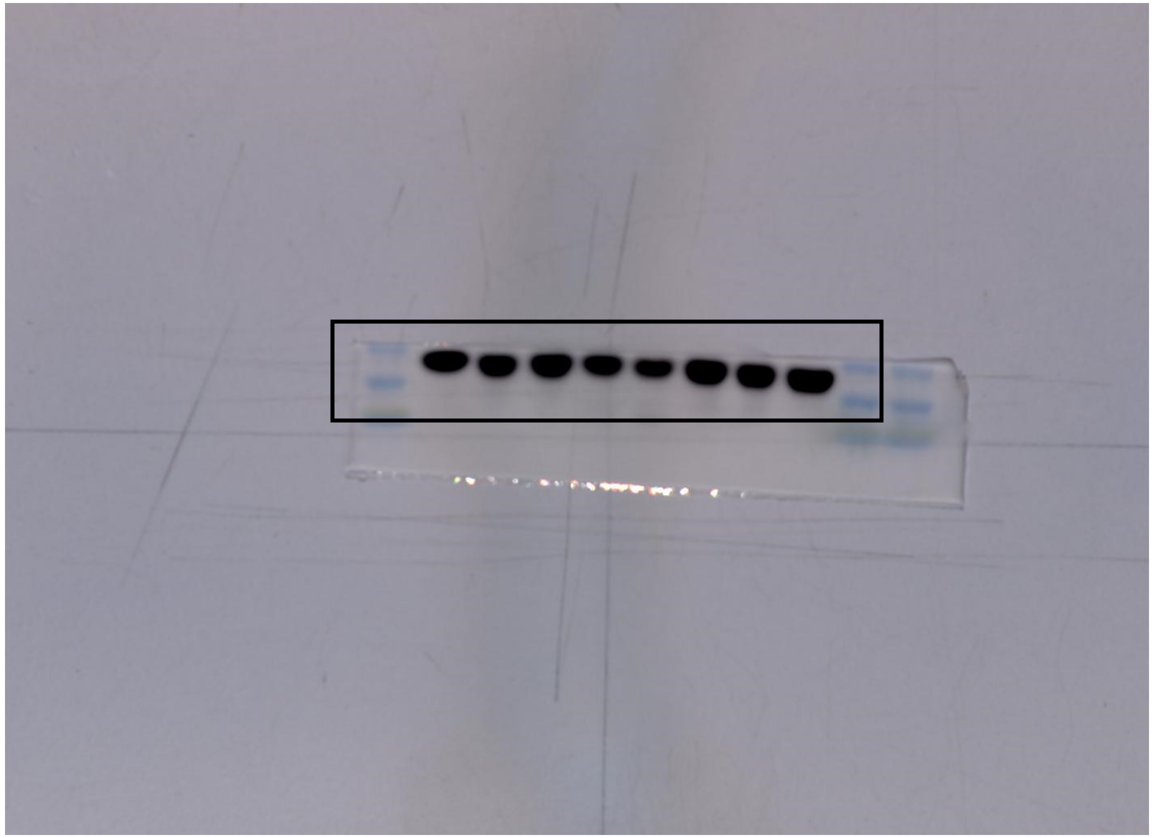

Figure 3E-PARP1

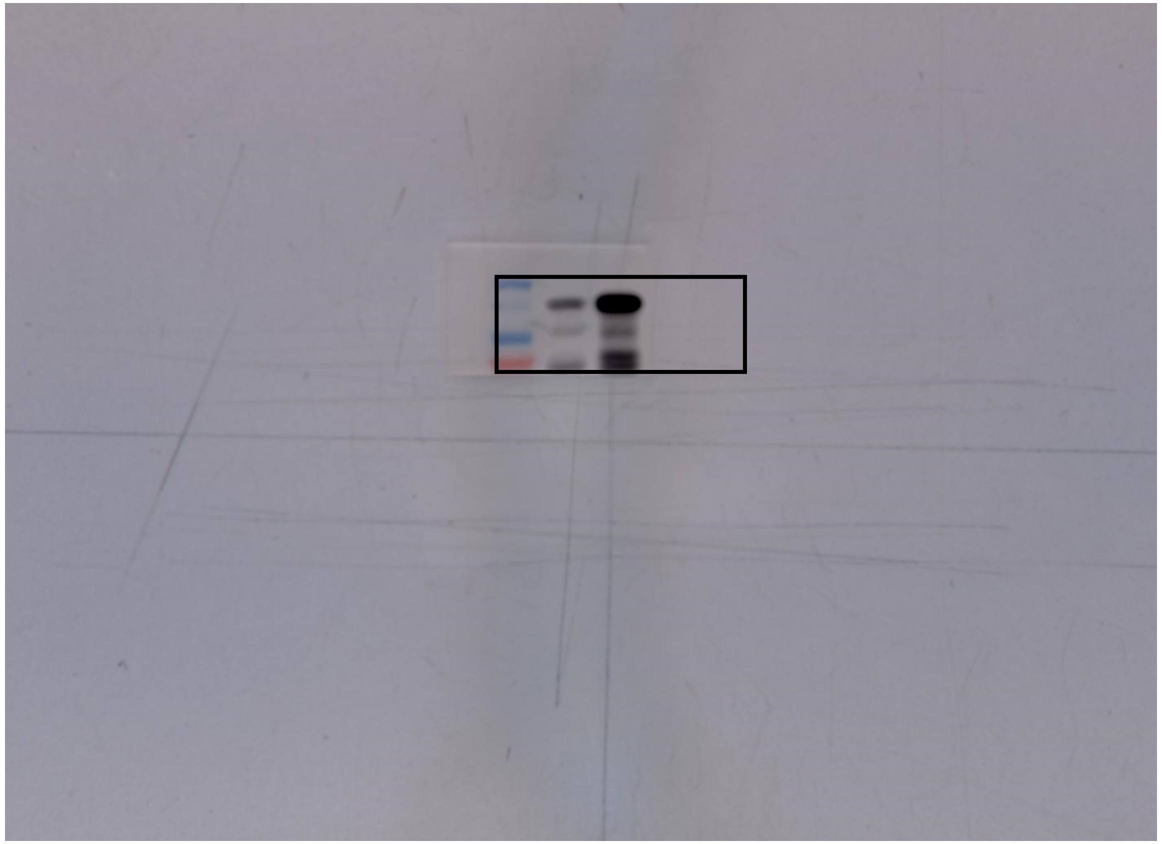

Figure 3E-H2A

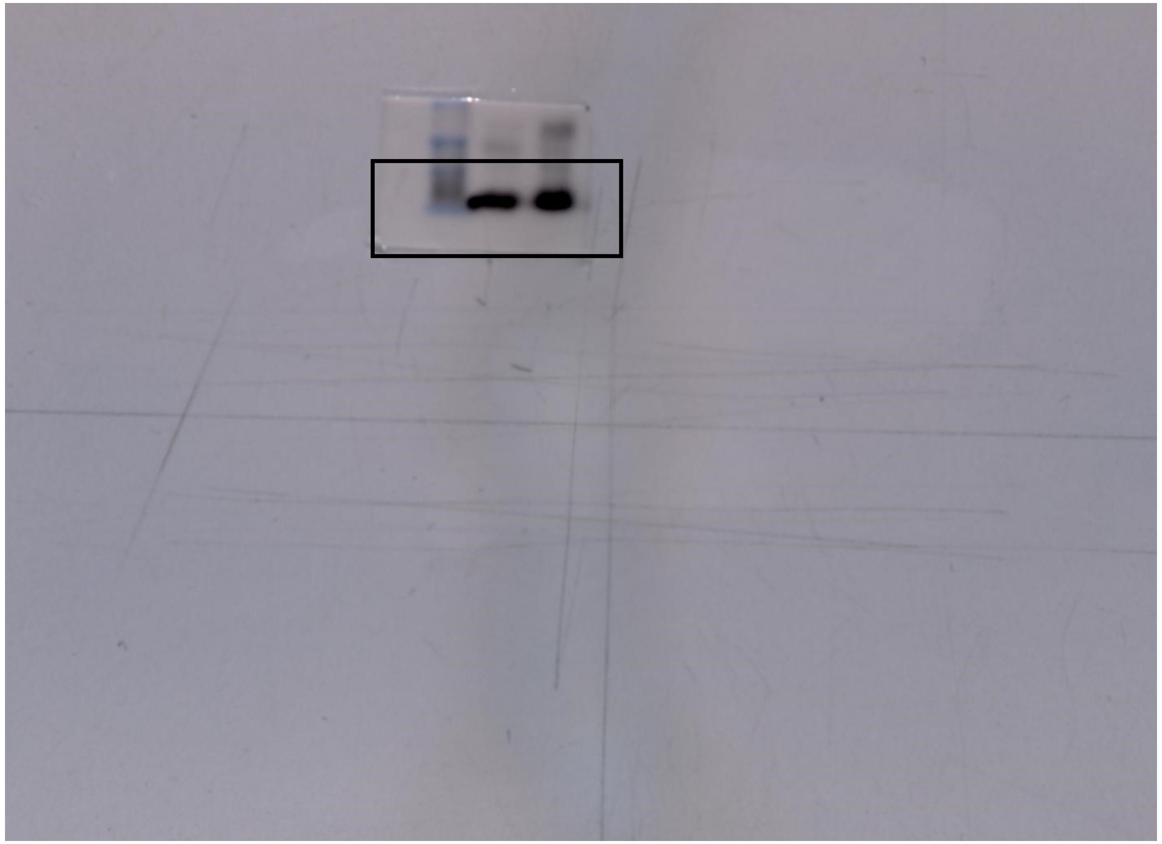

Figure 3E-PAR

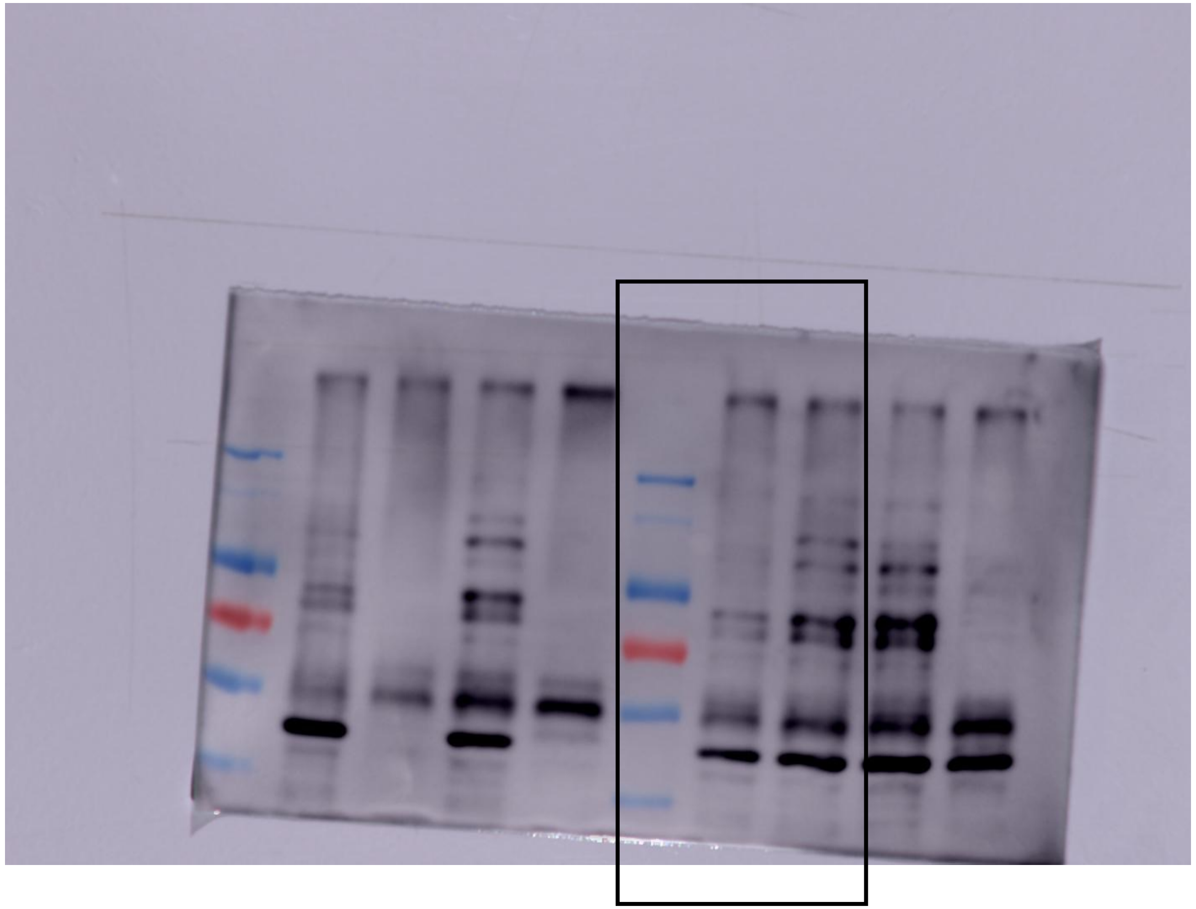

Figure 3E-β-actin

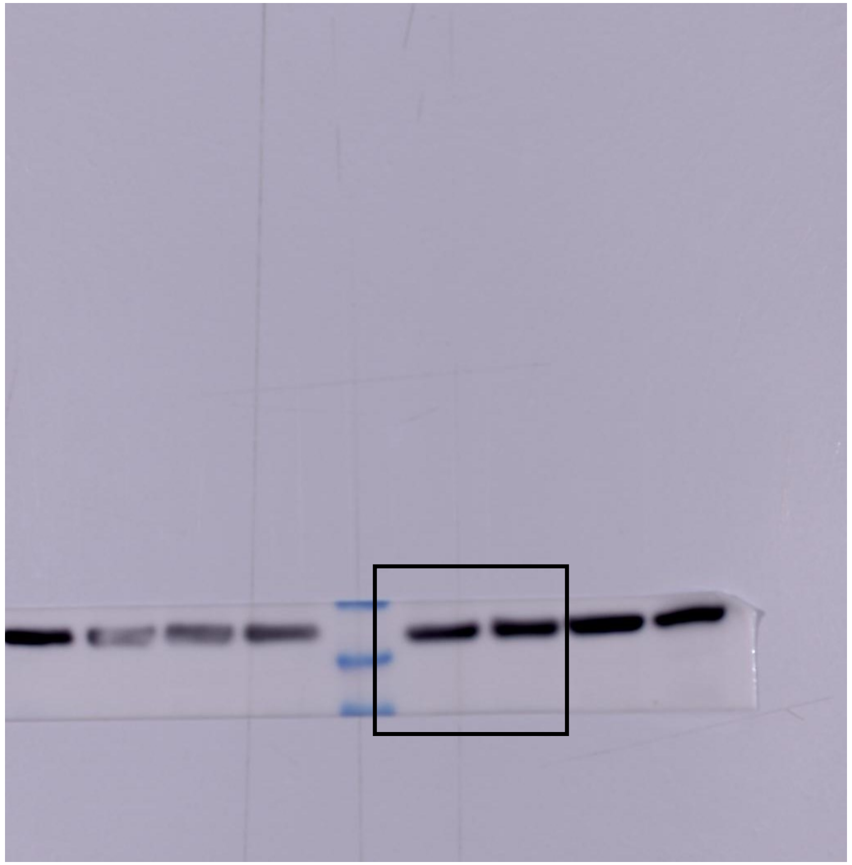

Figure 4E-PARP1

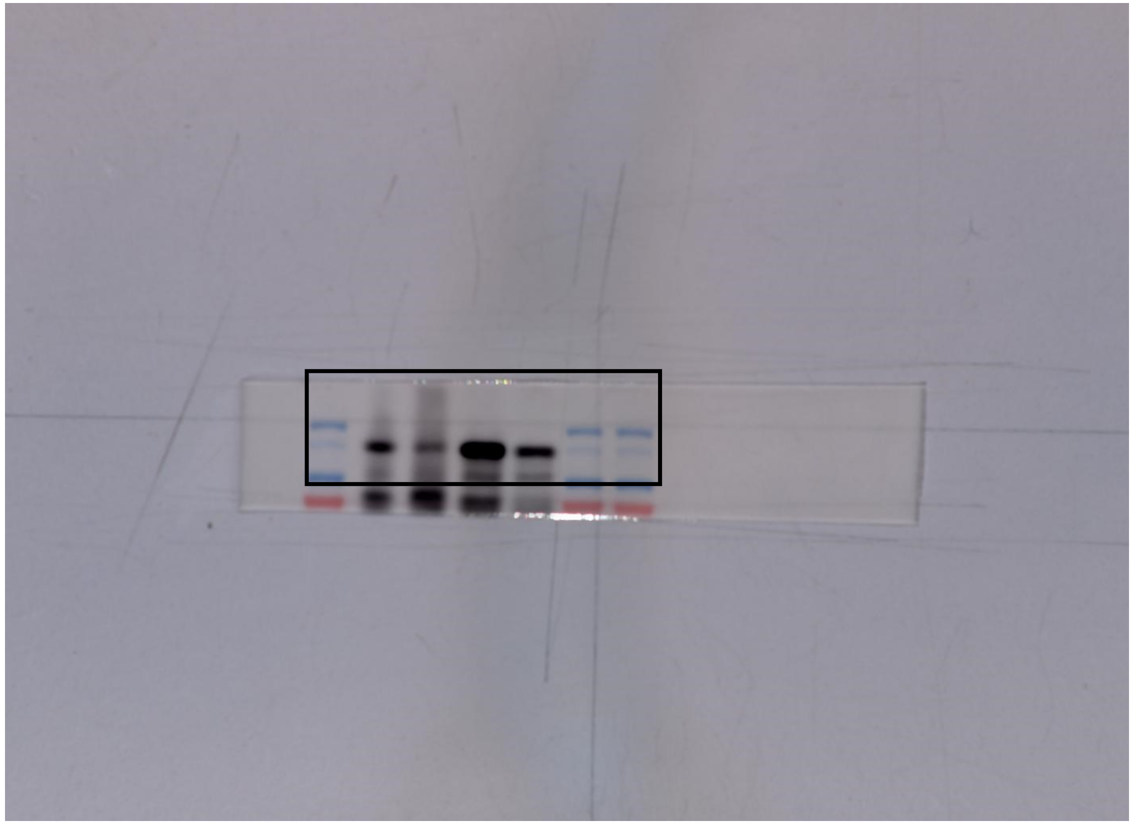

Figure 4E-H2A

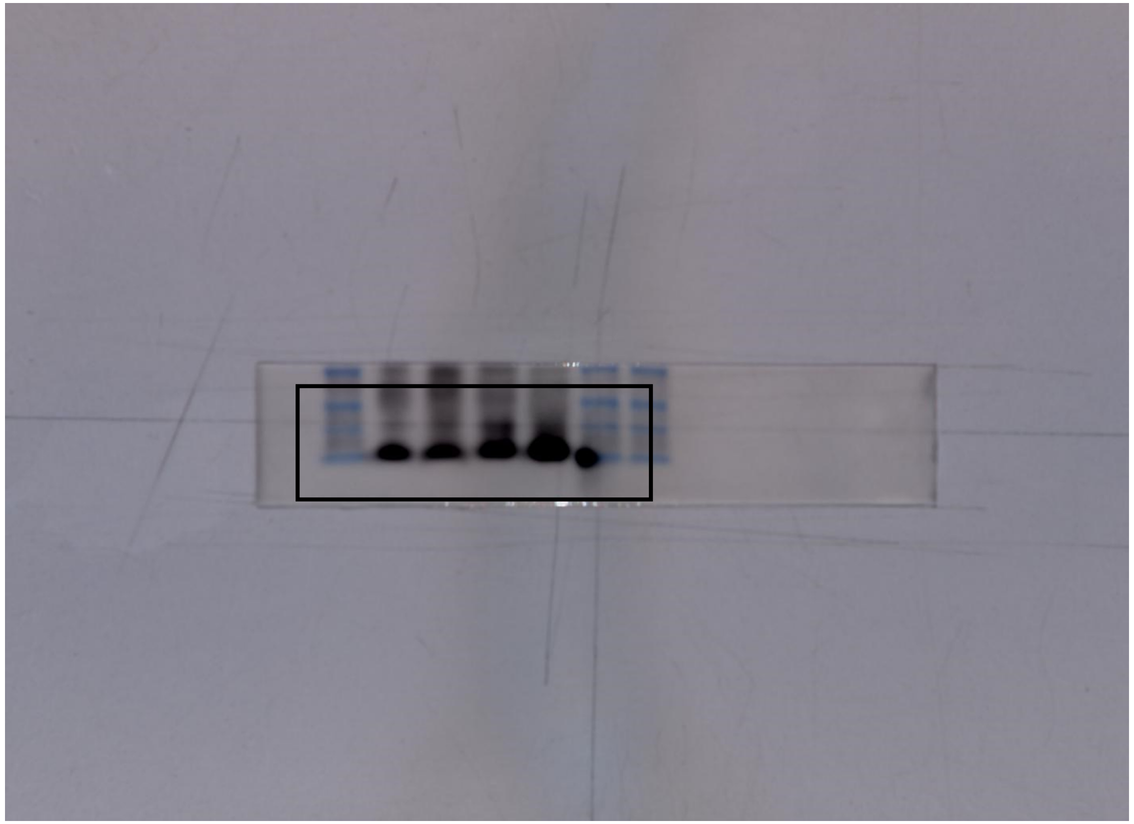

Figure 4E-PAR

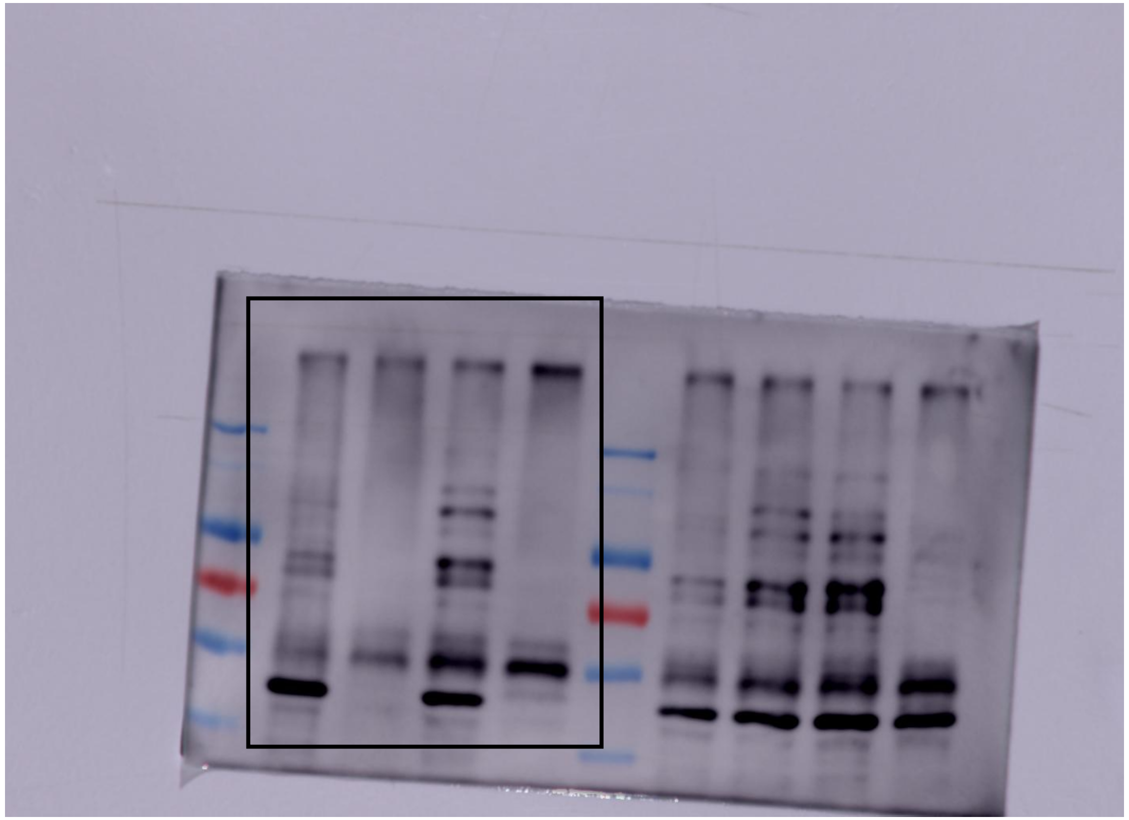

Figure 4E-β-actin

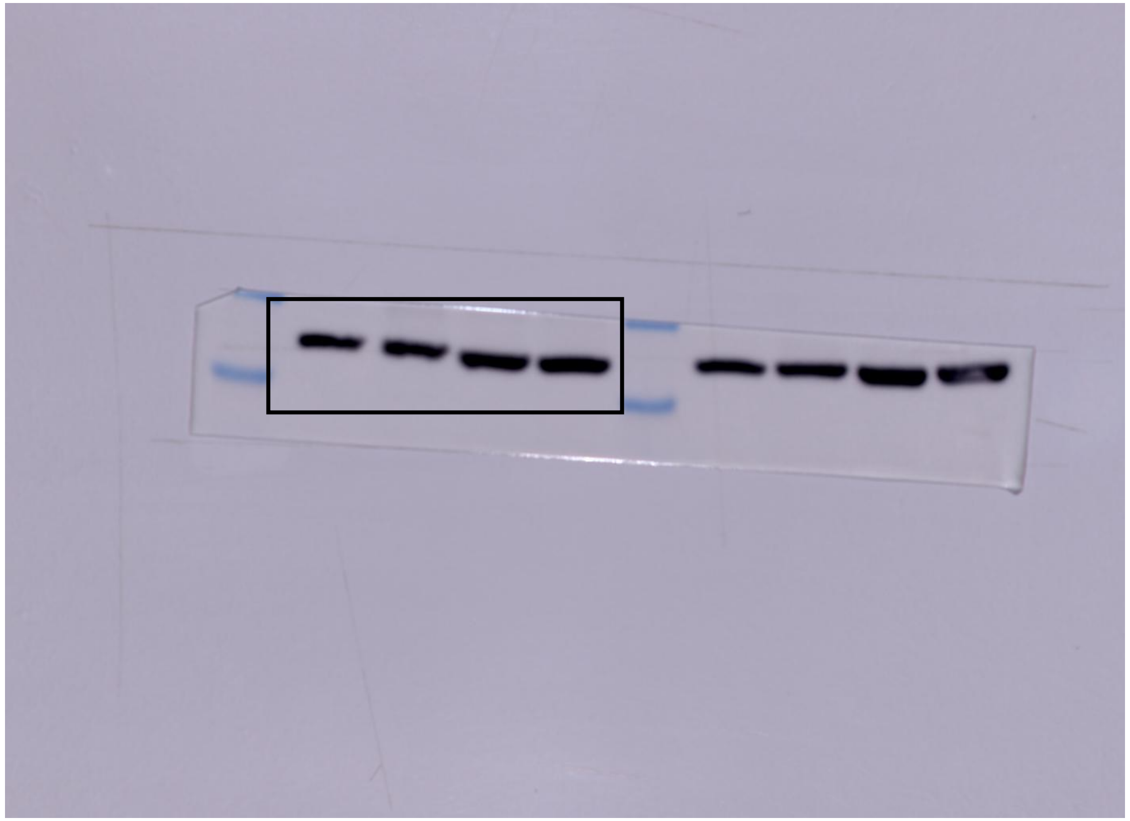

Figure 5F-E-cadherin

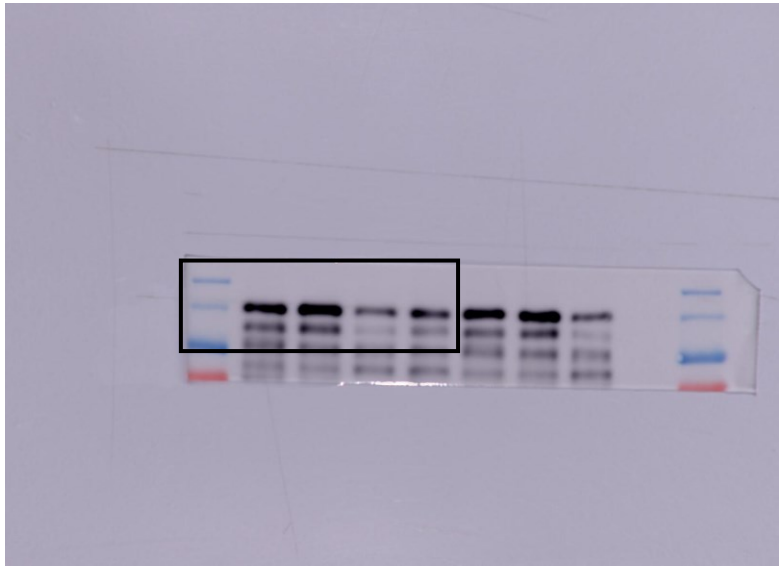

Figure 5E-β-catenin

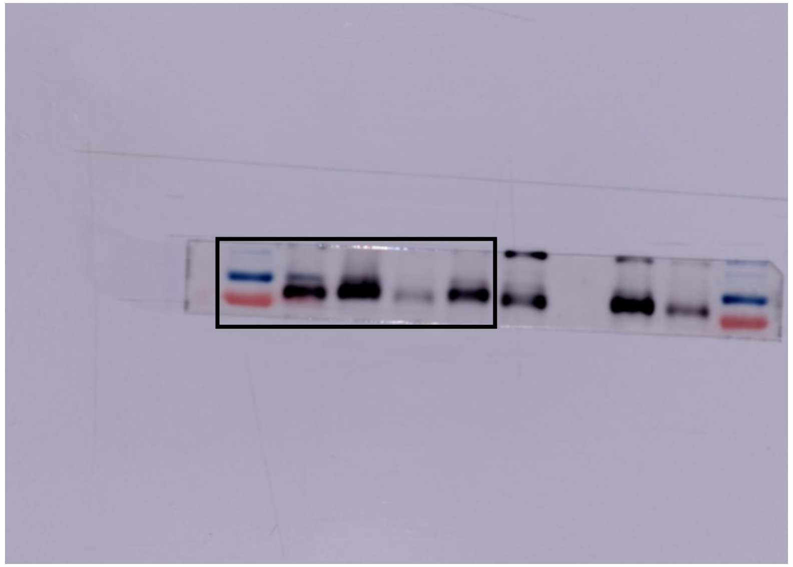

Figure 5E-β-actin

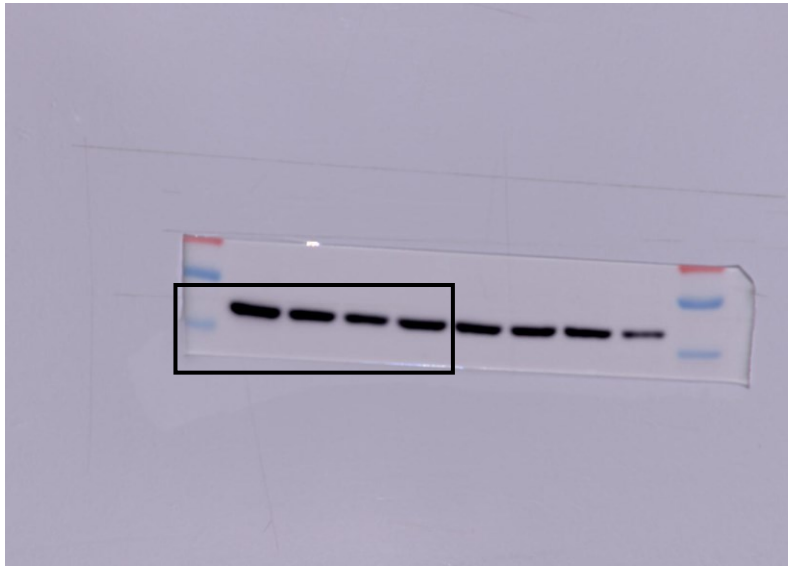

Figure 6A-PARP1

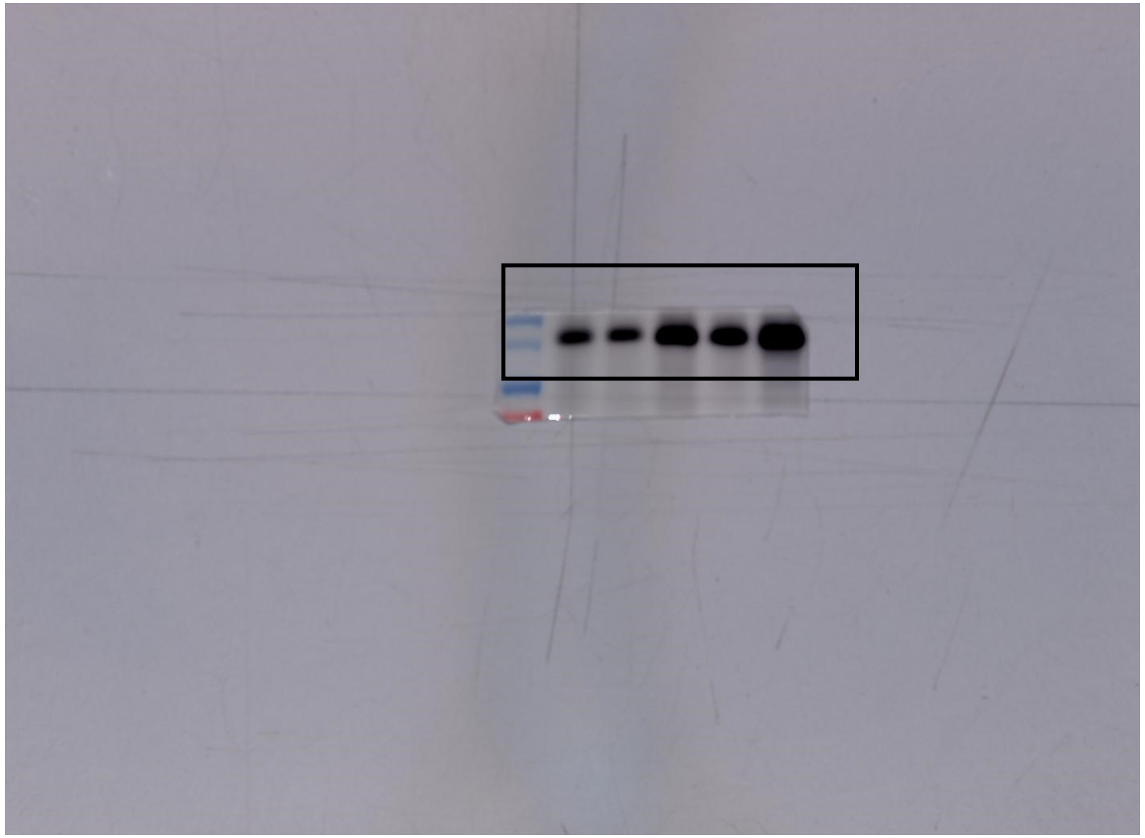

Figure 6A-H2A

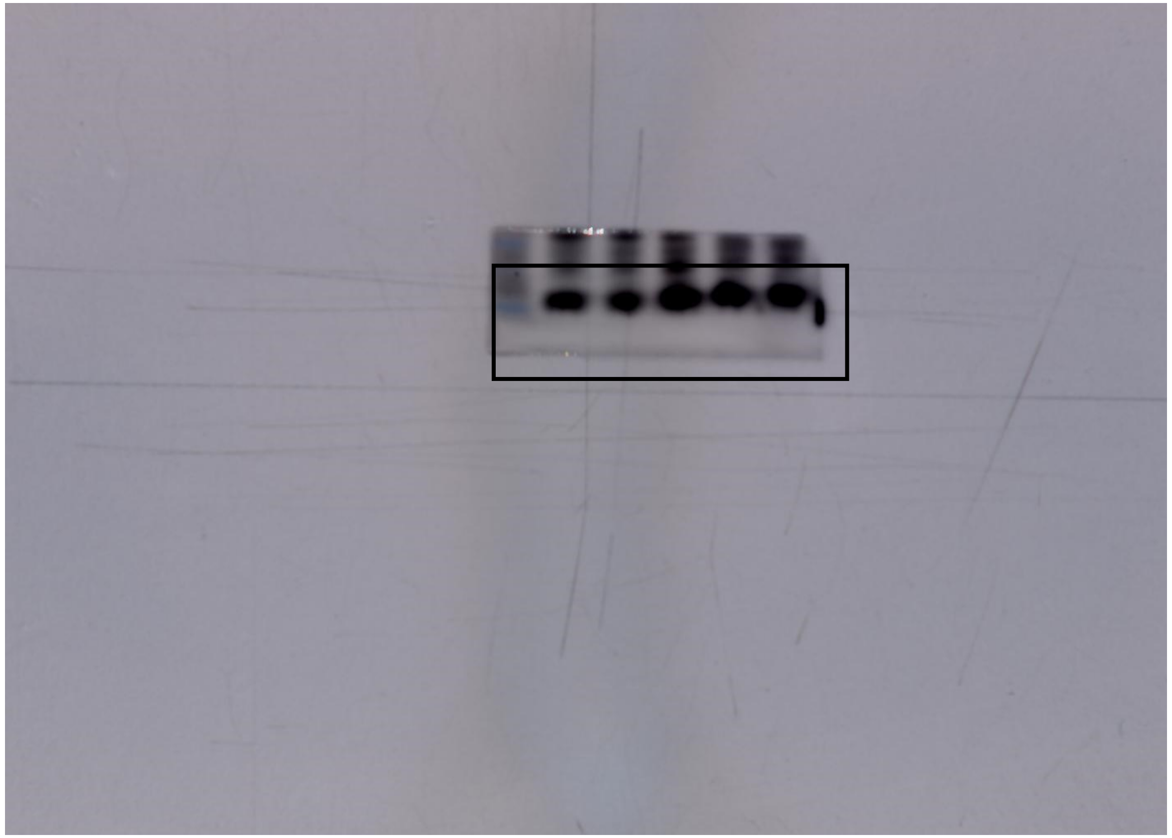

Figure 6A-PAR

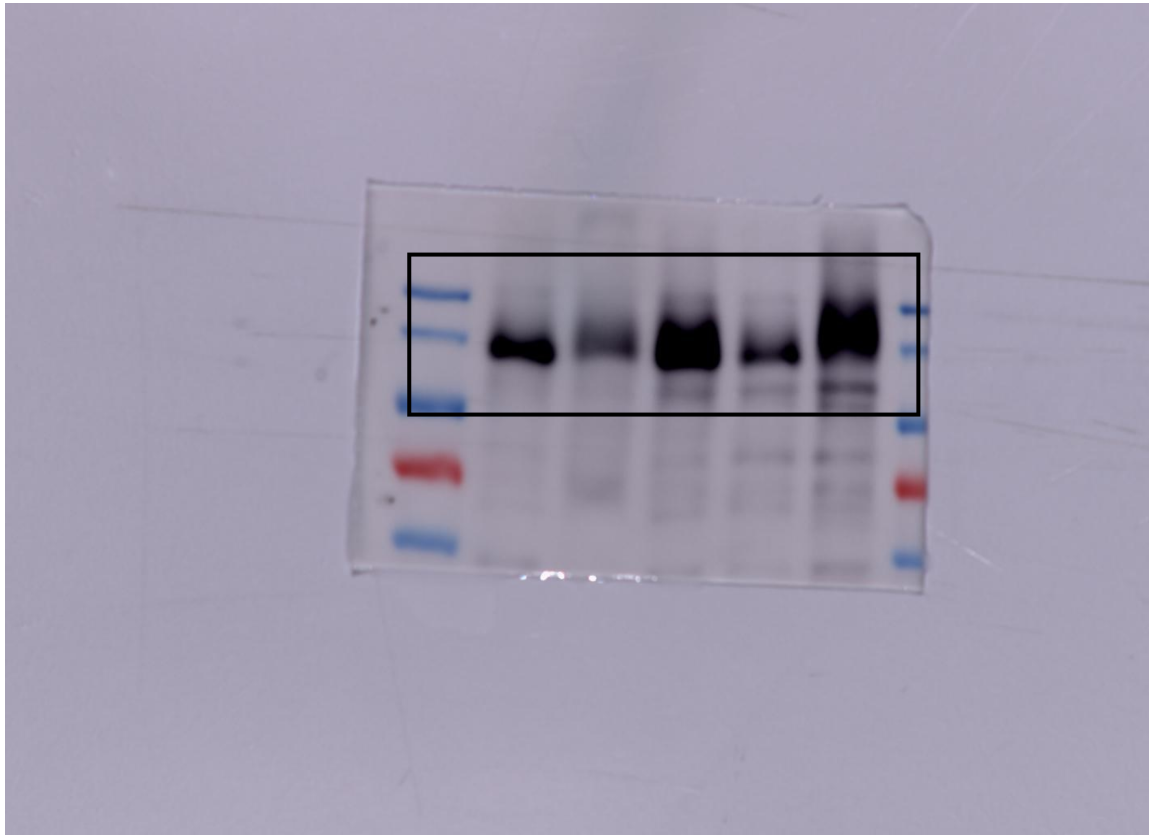

Figure 6A-β-actin

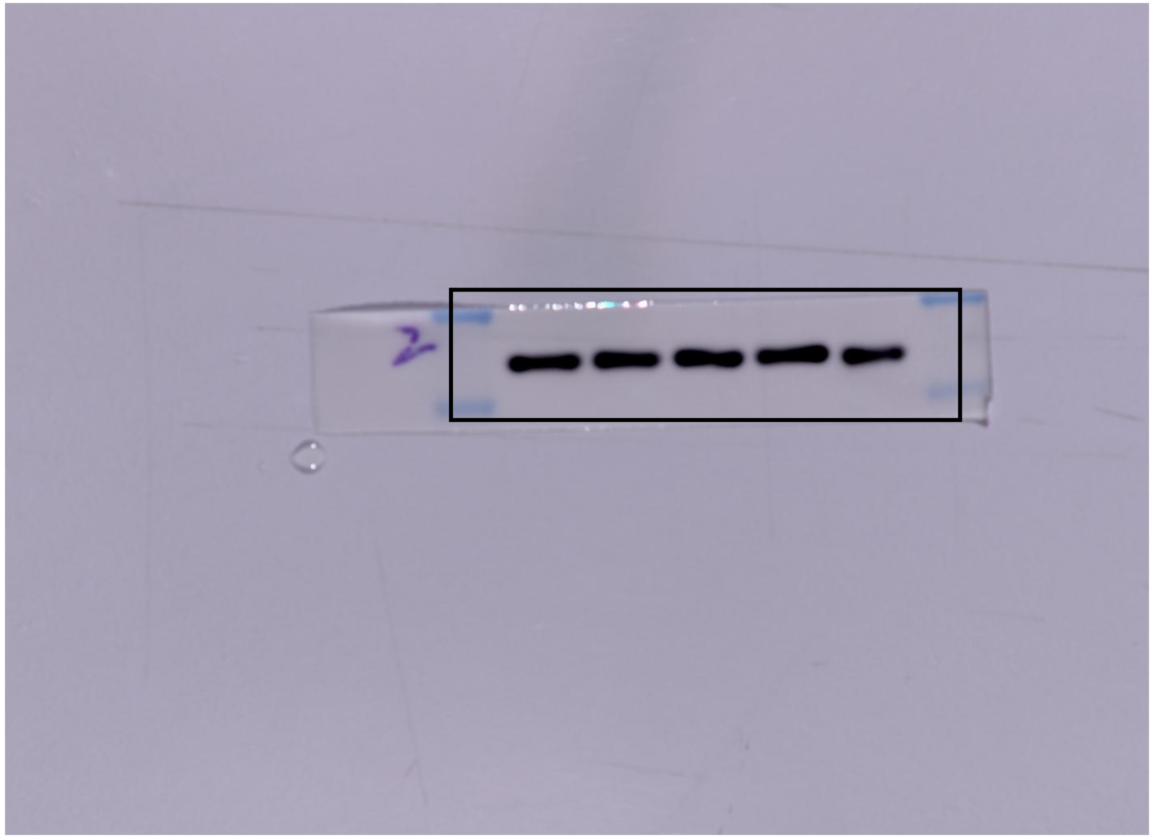

Figure 6G-AIF

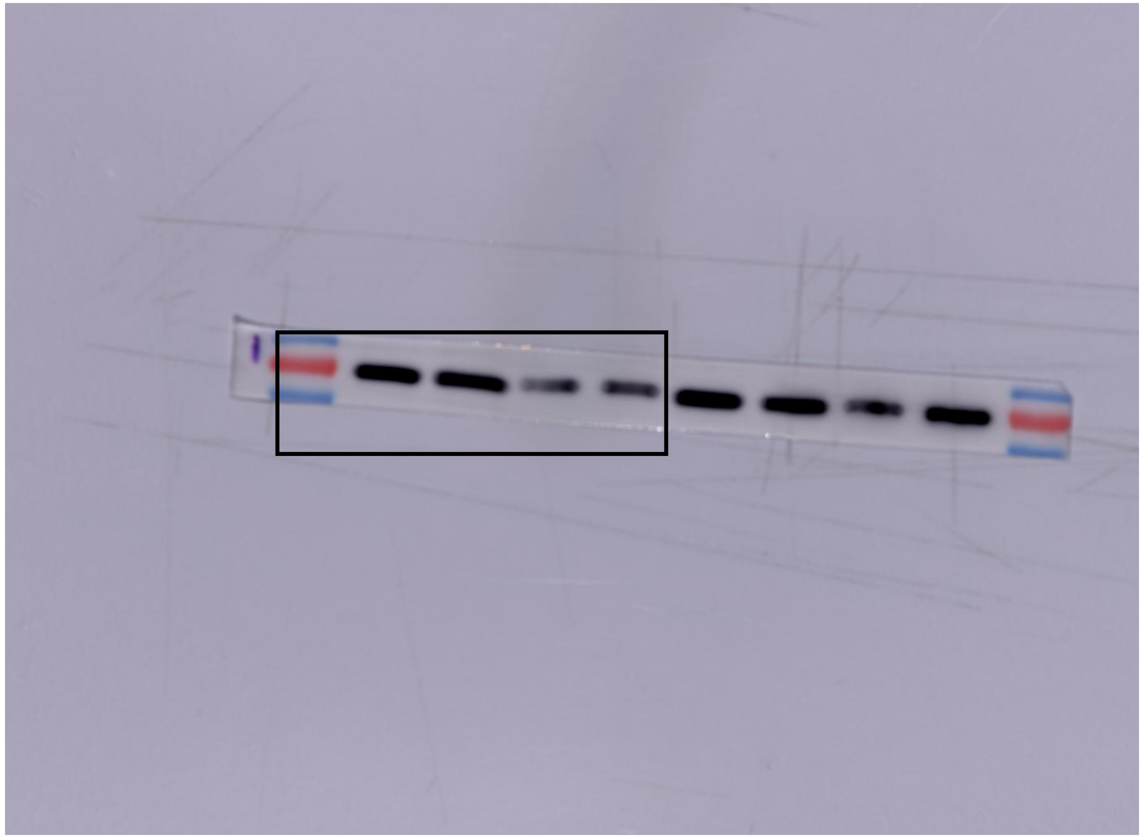

Figure 6G-COX-IV

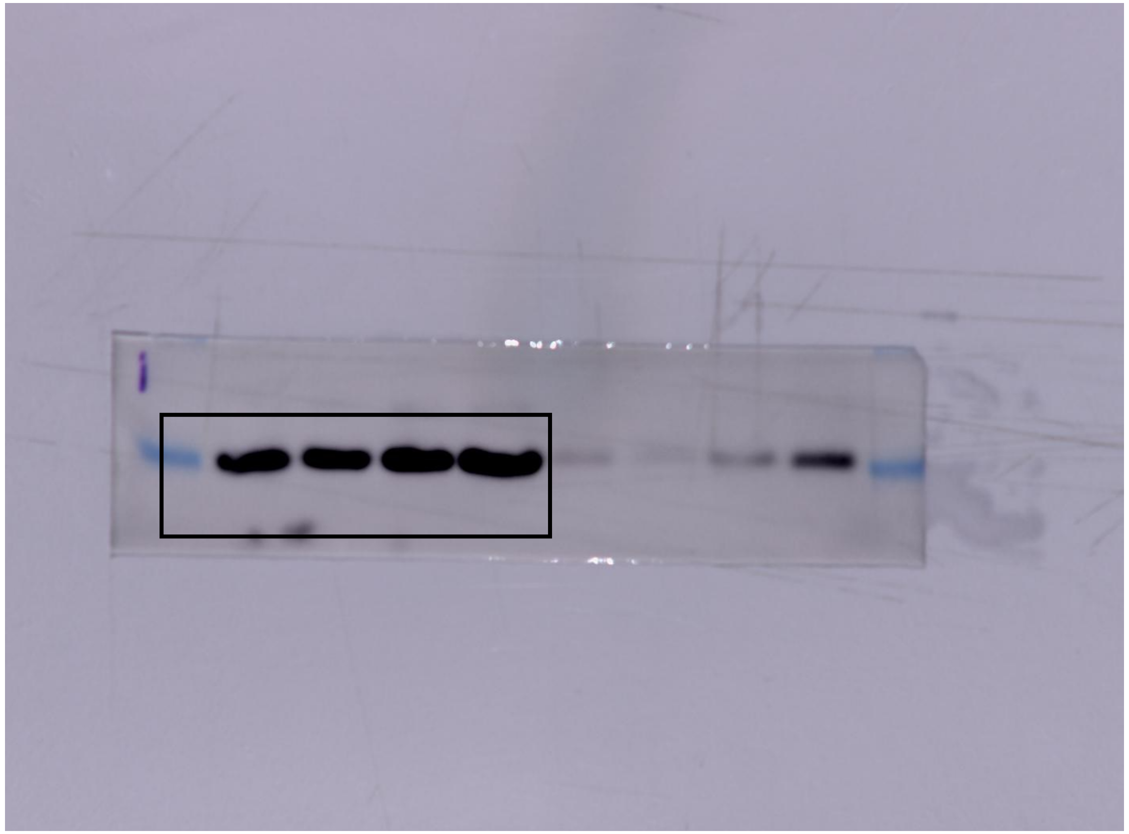

Figure 6H-AIF

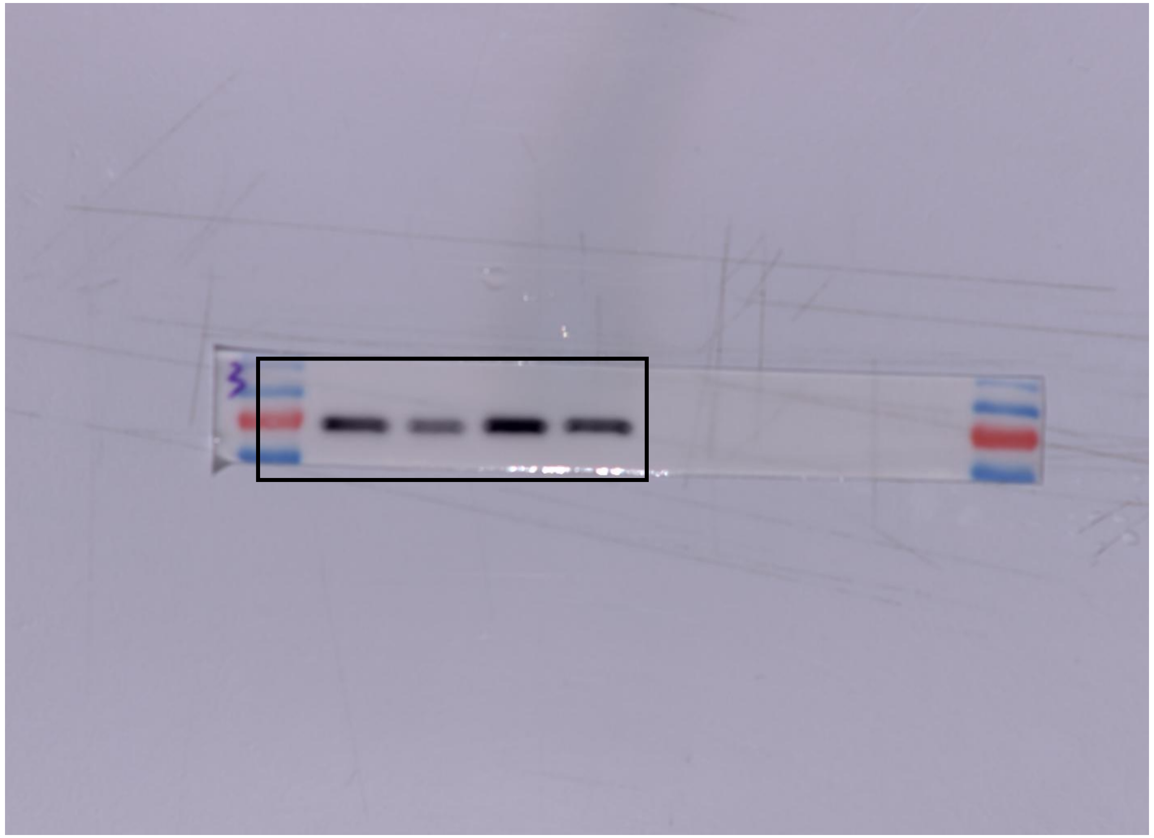

Figure 6H-β-actin

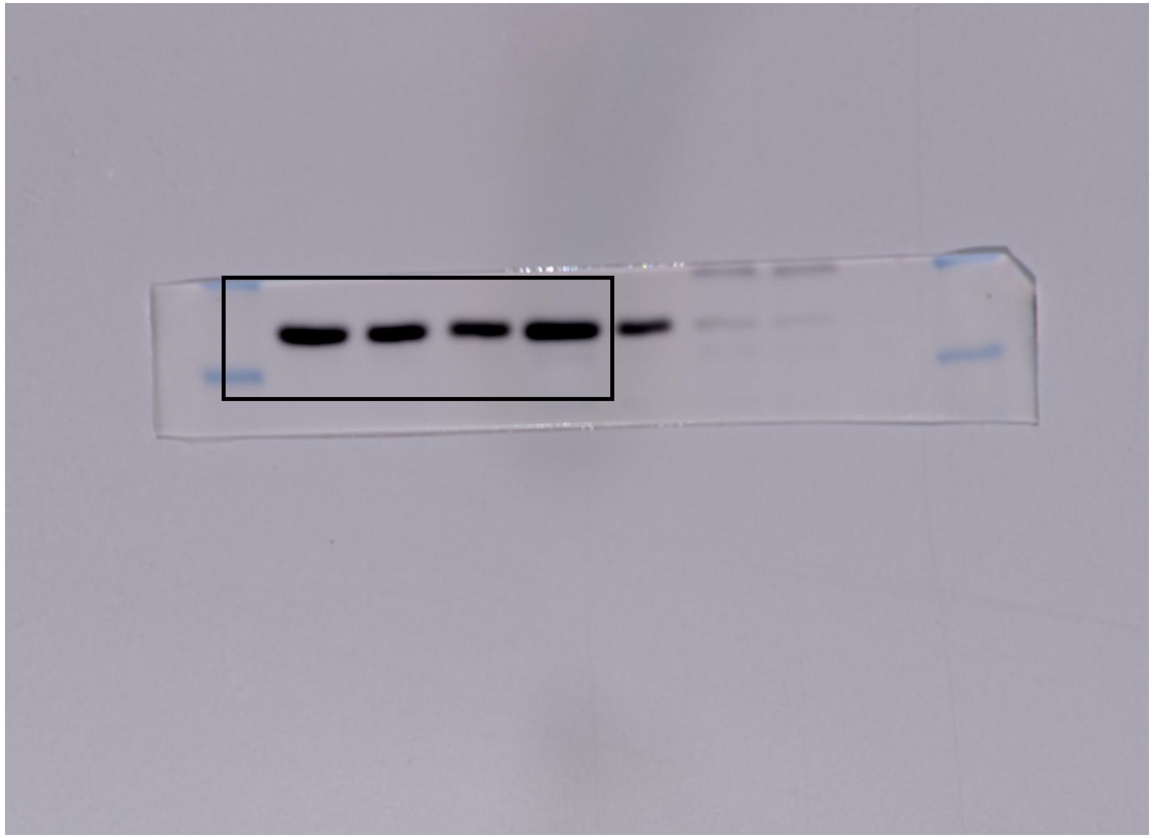

Figure 6J-AIF

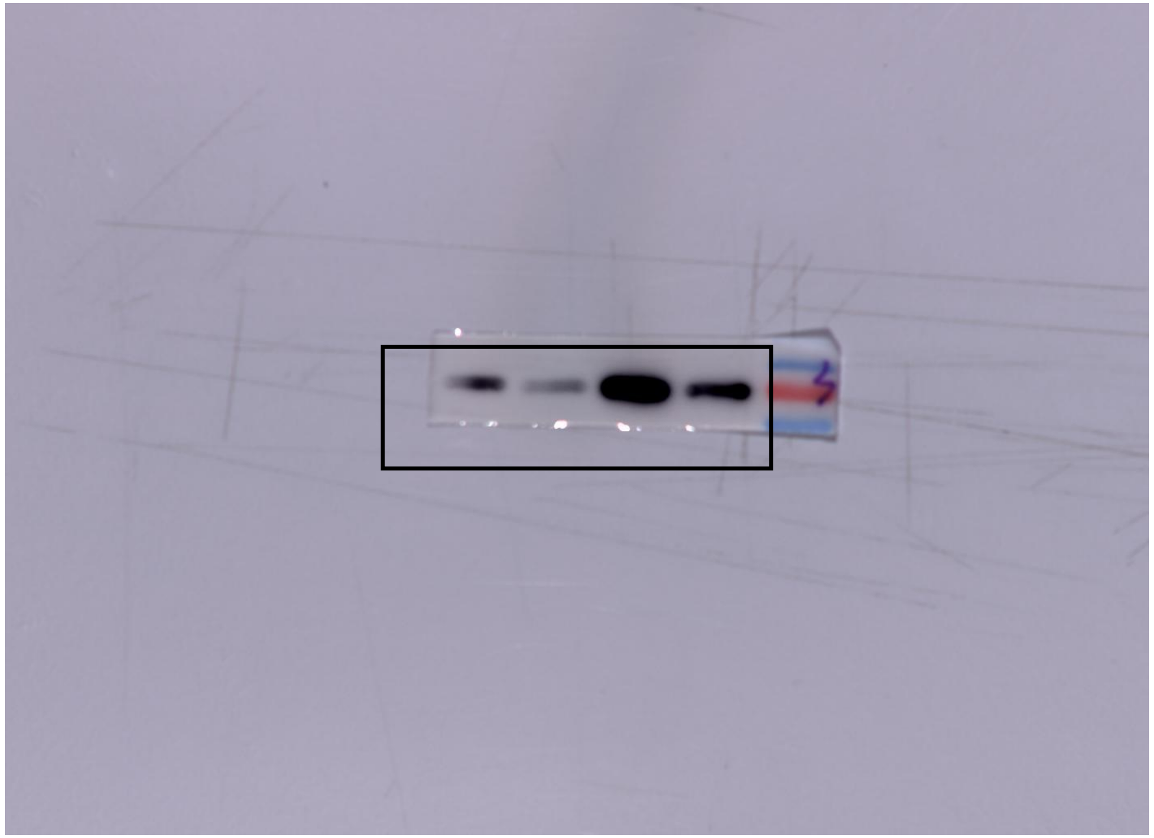

Figure 6J-Histone-H3

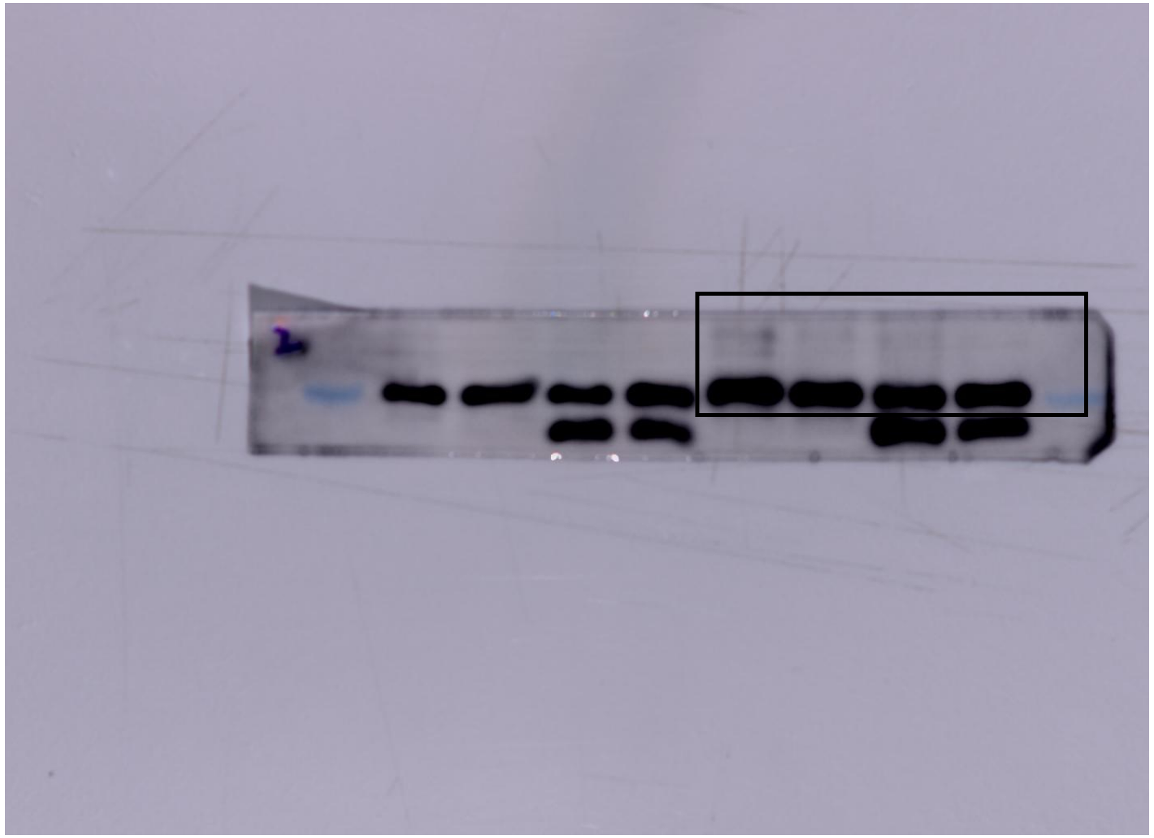

Figure 7F-PARP1

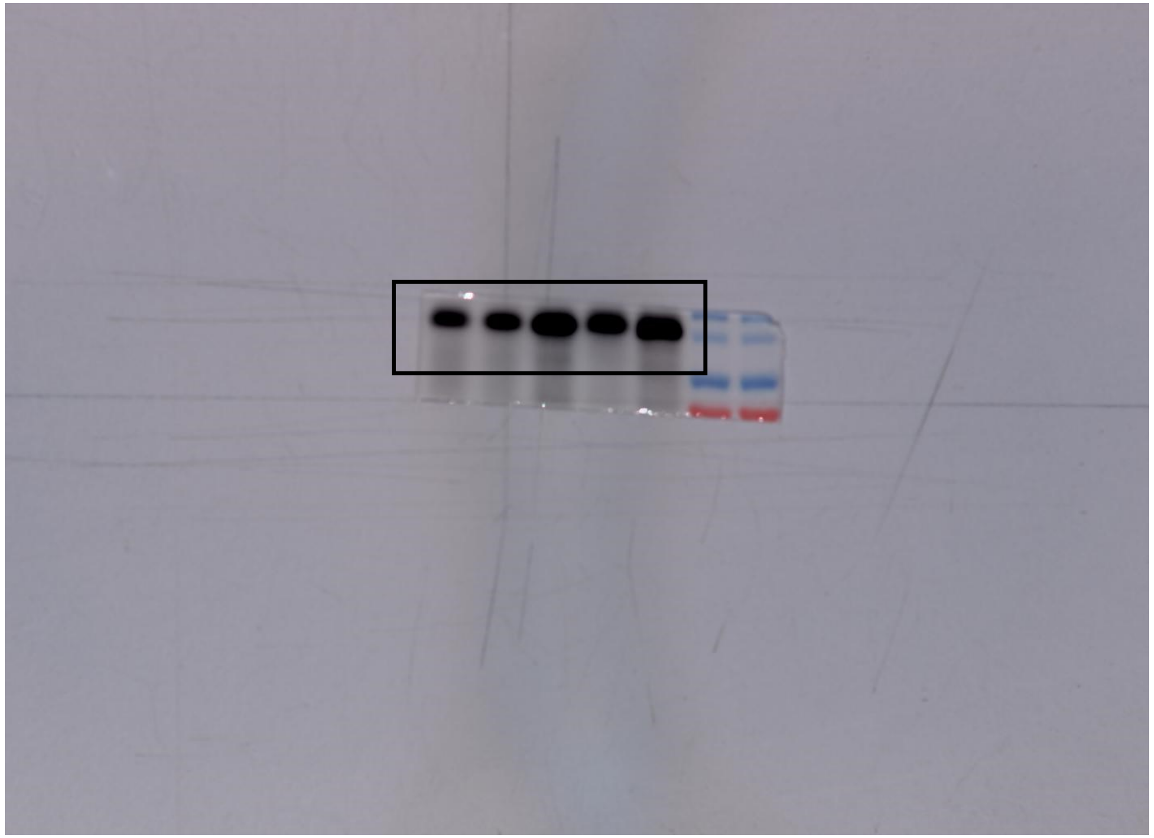

Figure 7F-H2A

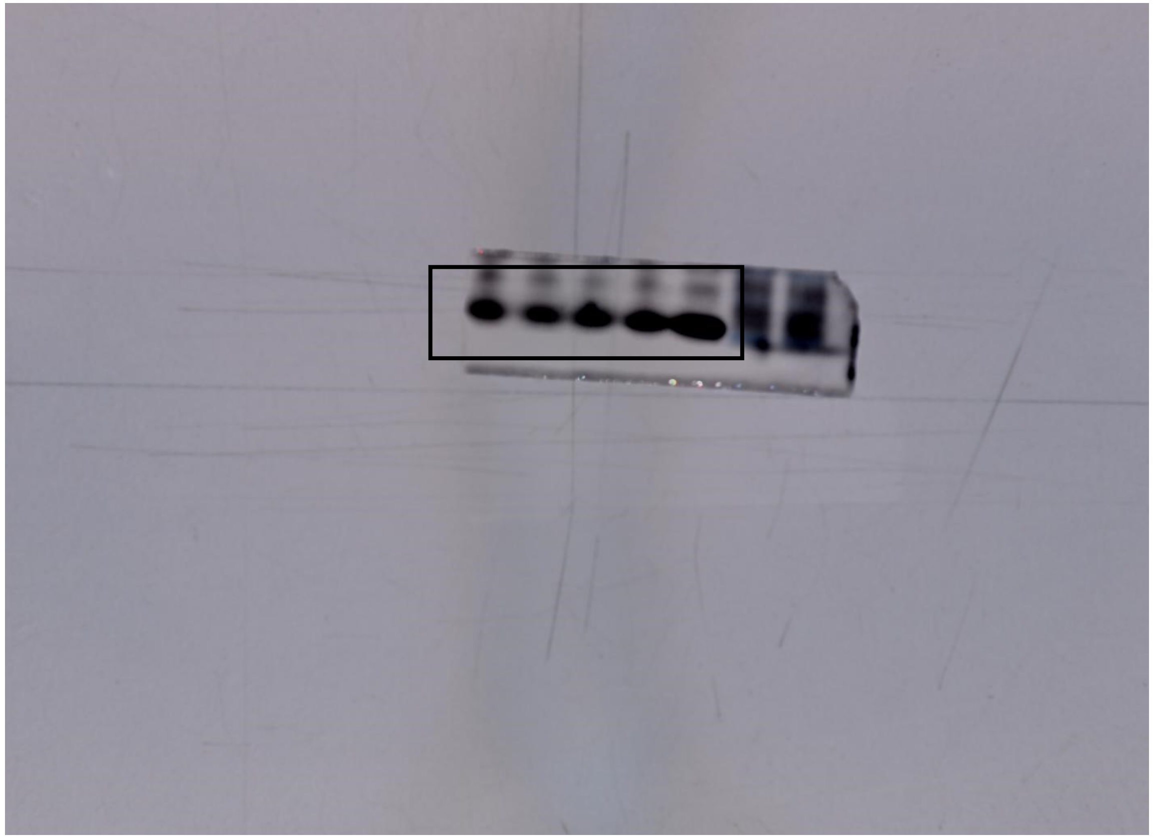

Figure 7F-PAR

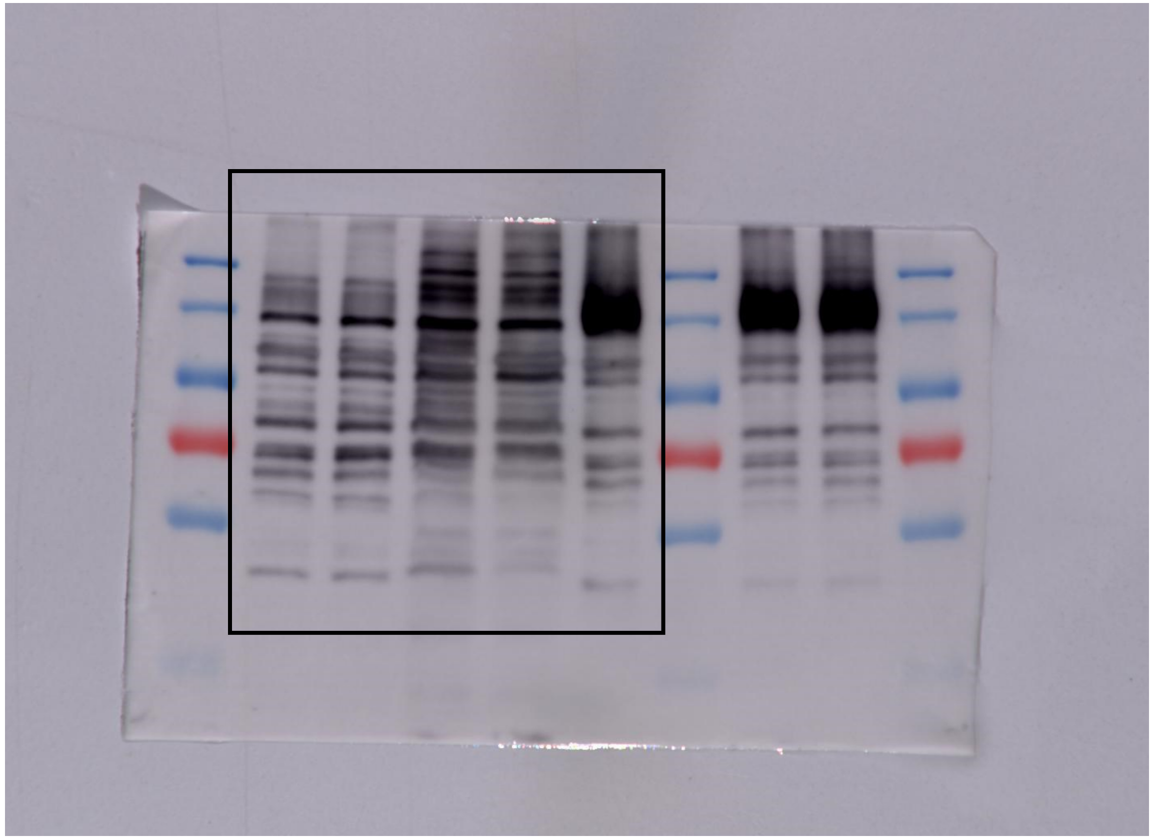

Figure 7F-β-actin

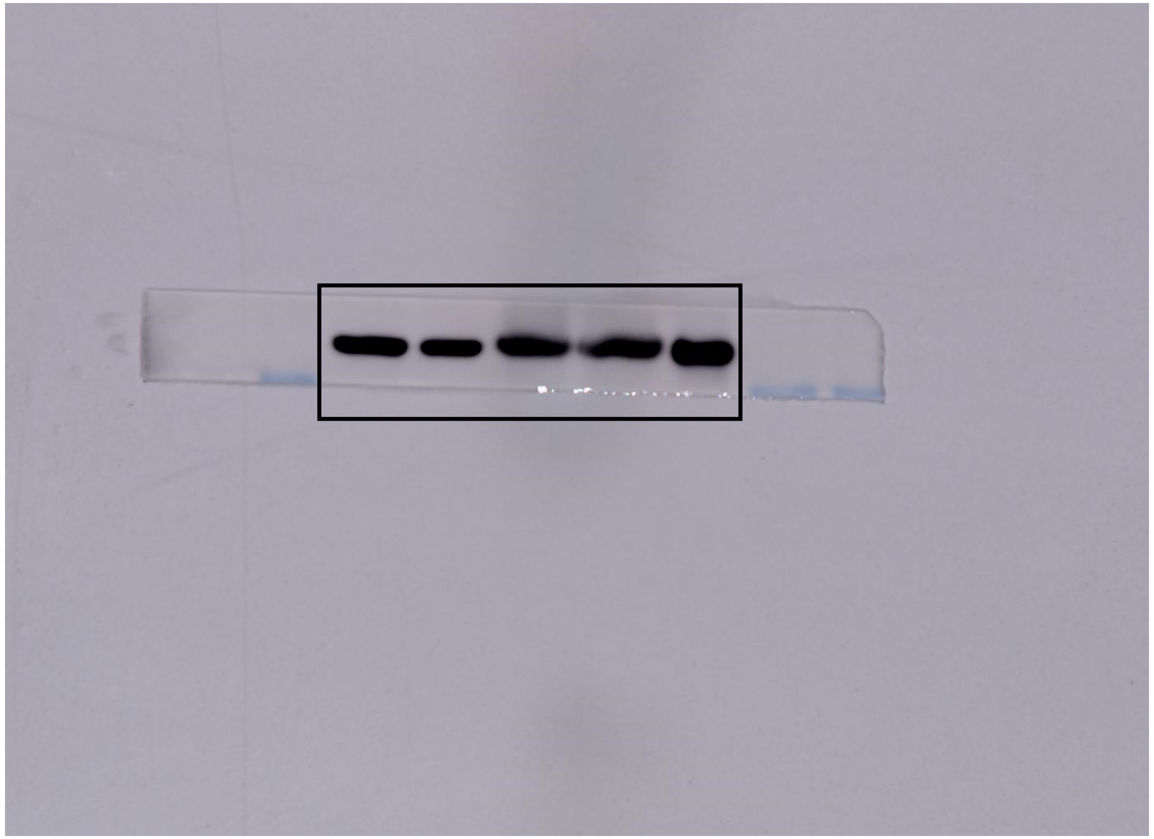

Figure 7I-AIF

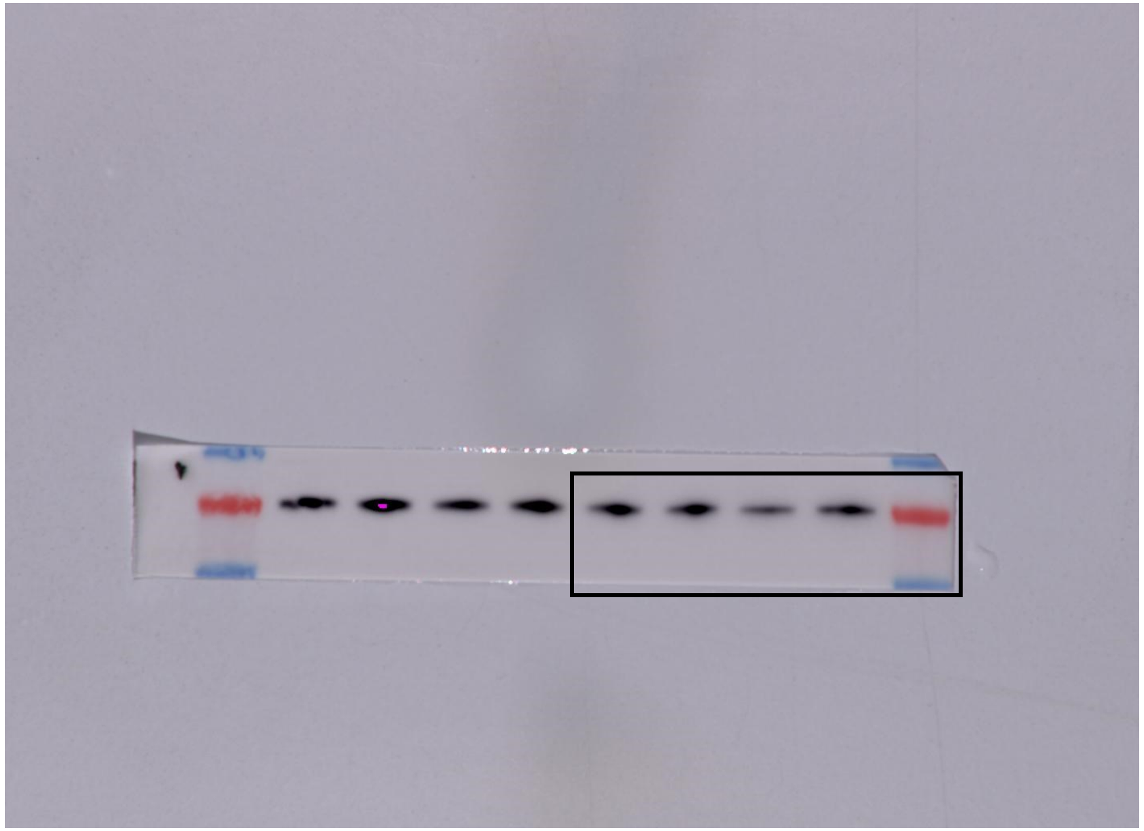

Figure 7I-COX-IV

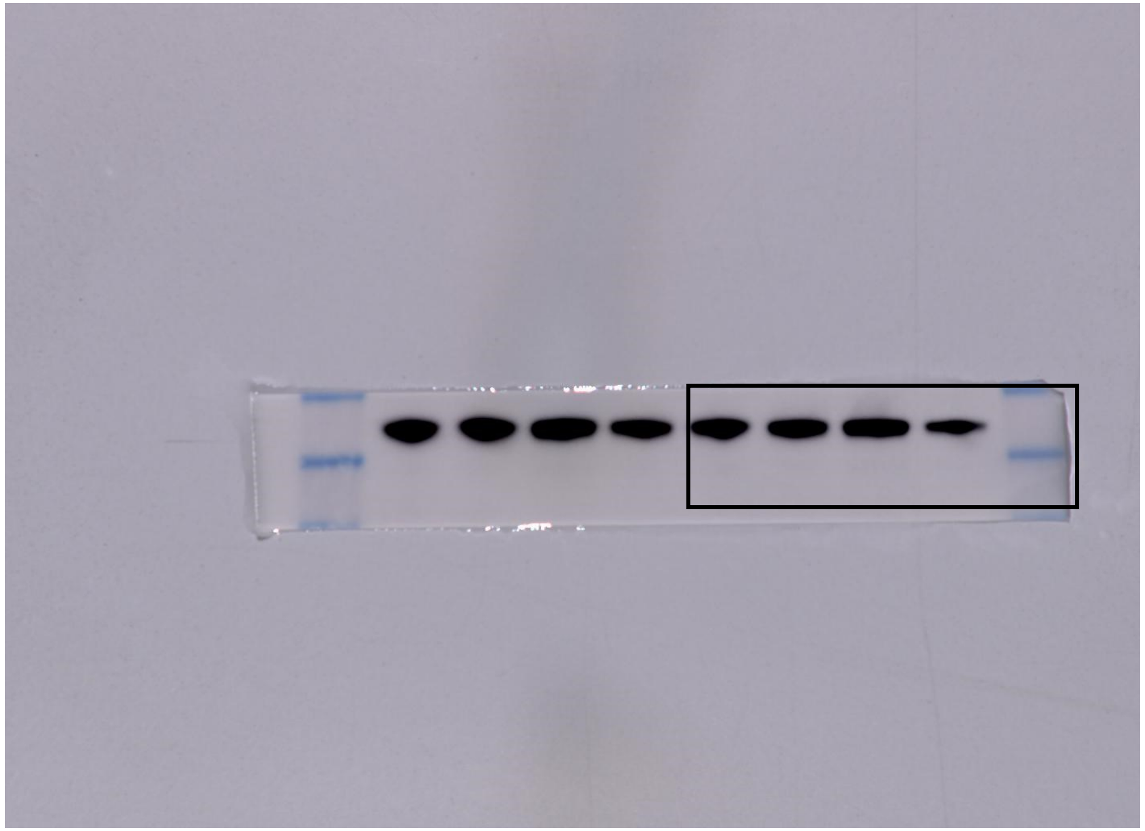

Figure 7J-AIF

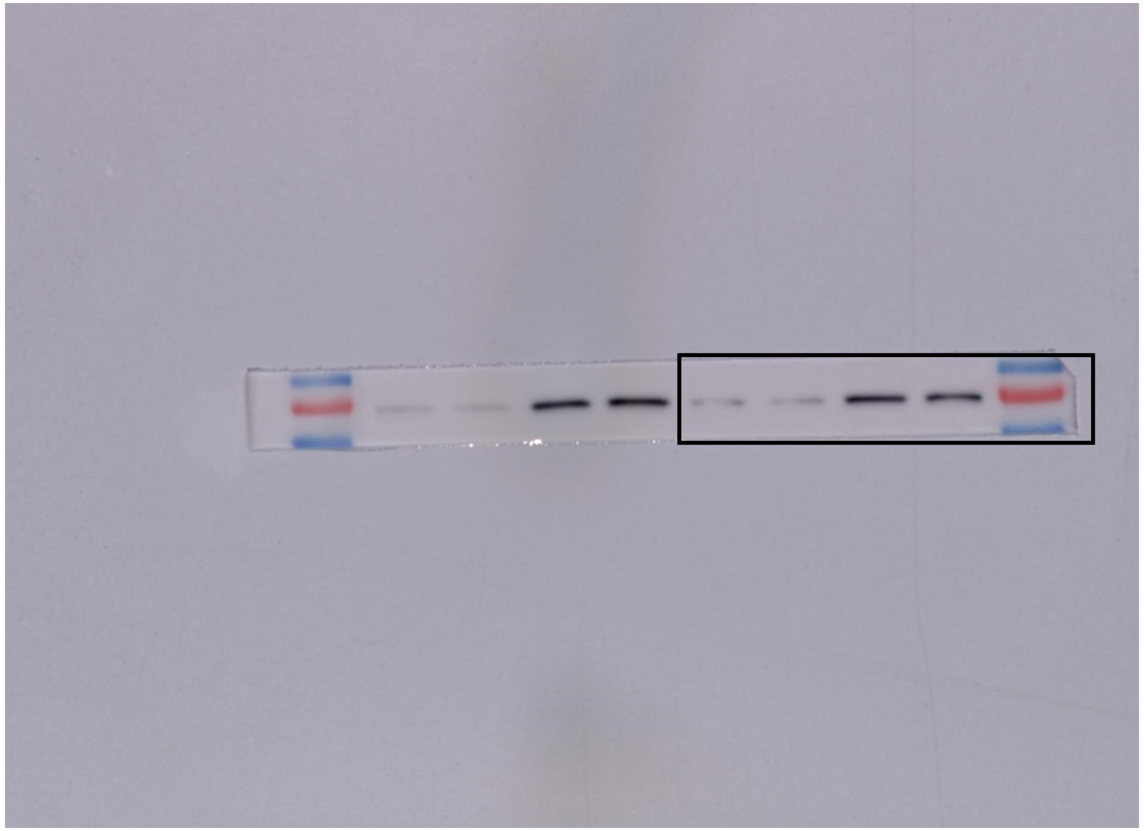

Figure 7J-β-actin

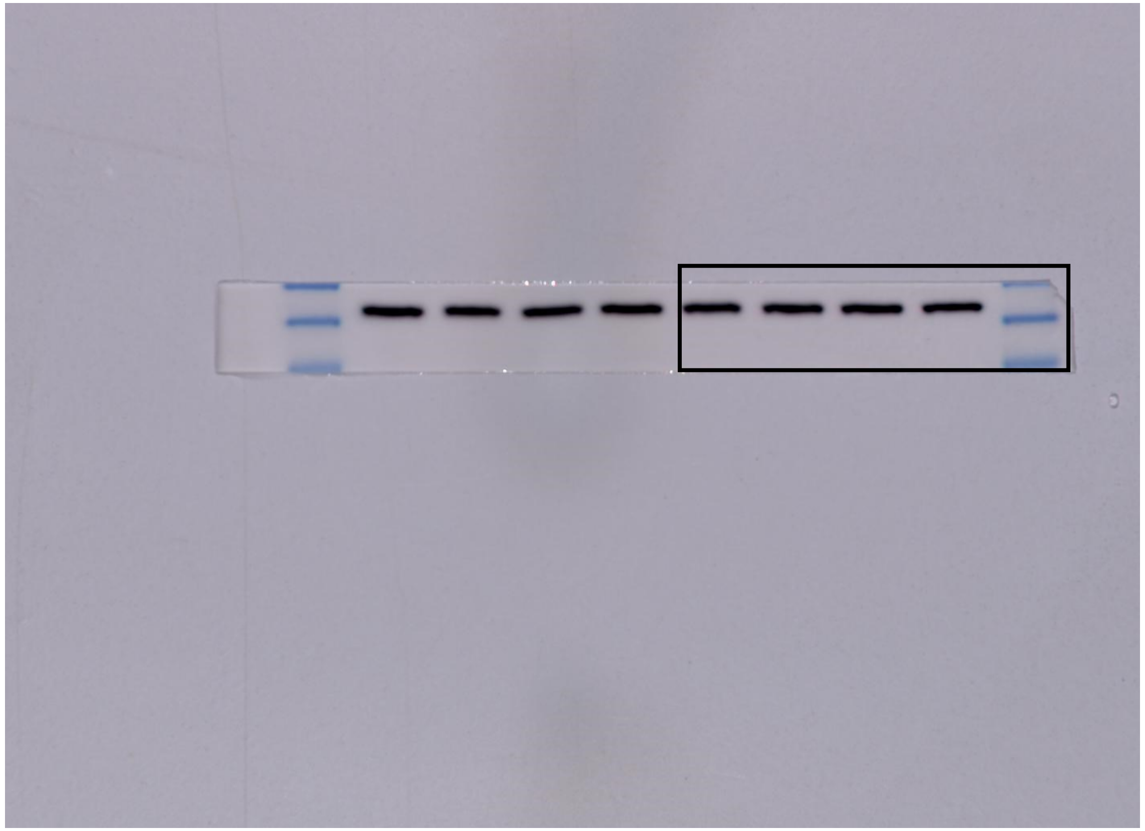

Figure 7K-AIF

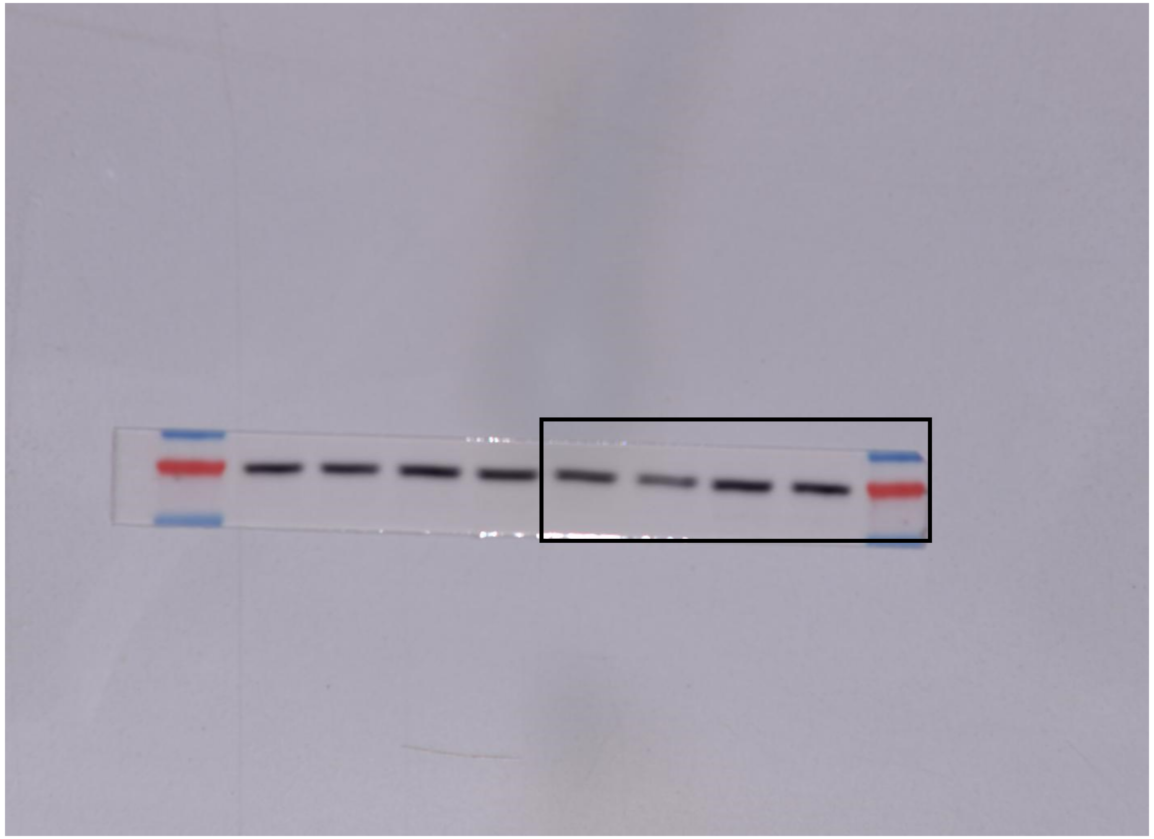

Figure 7K-Histone-H3

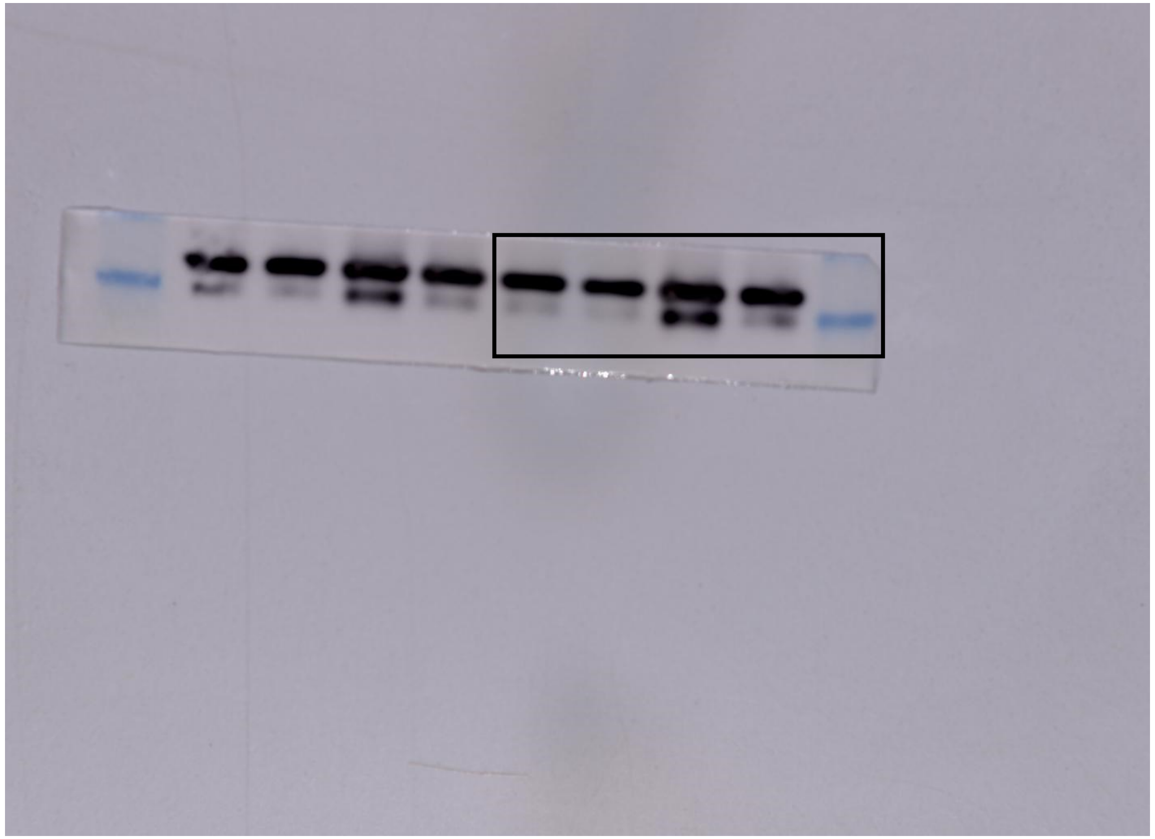

Supplement Figure 2A-PARP1

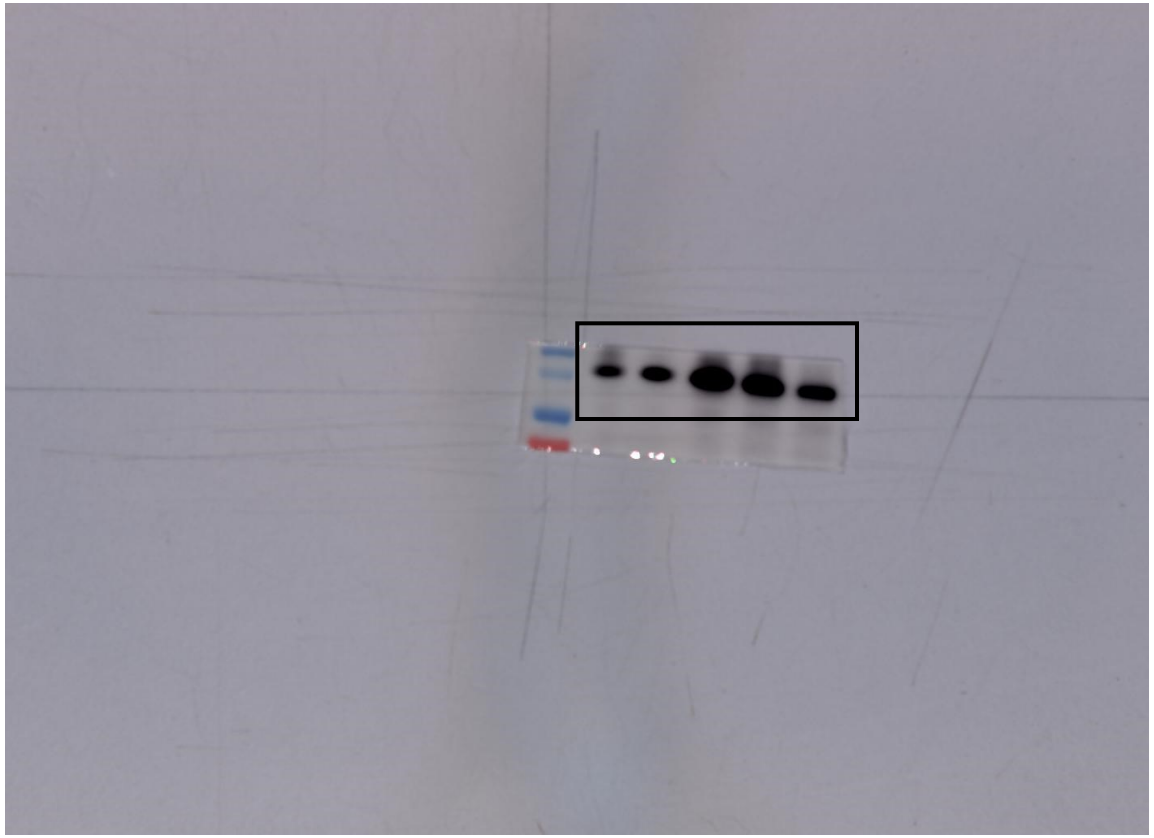

Supplement Figure 2A-H2A

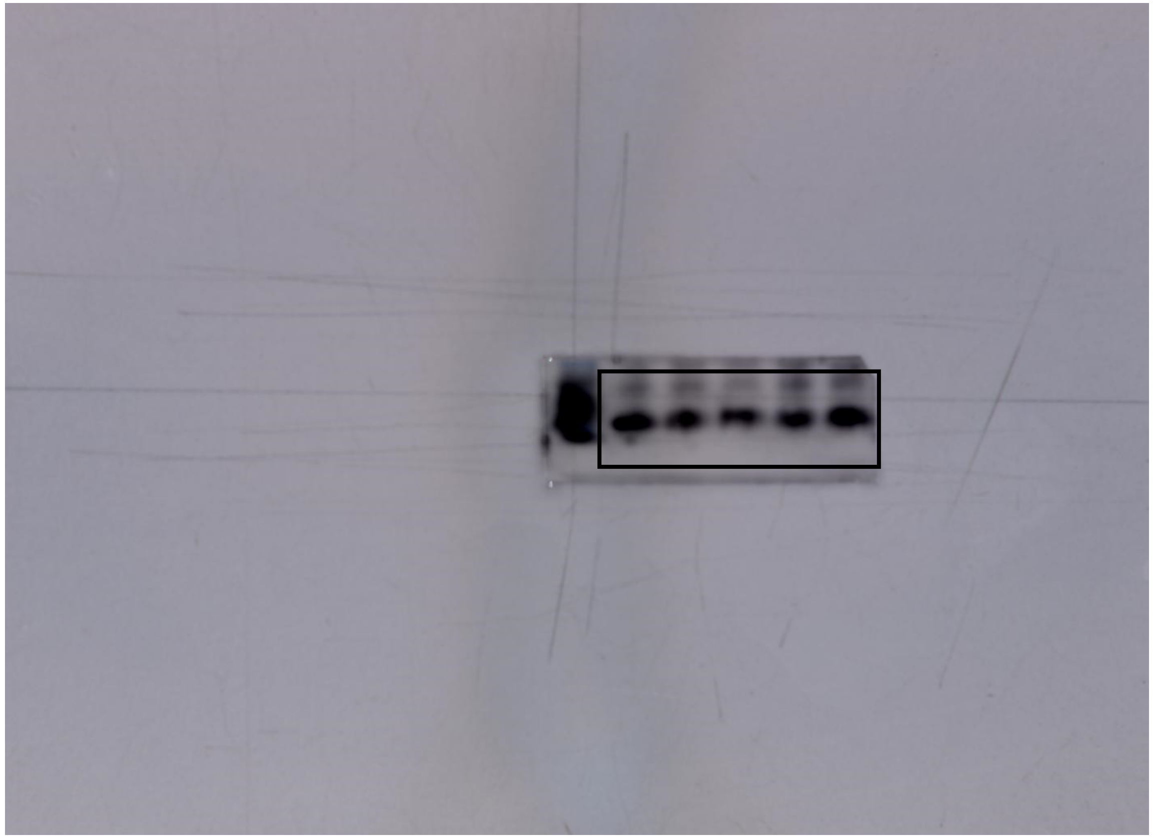

Supplement Figure 2A-PAR

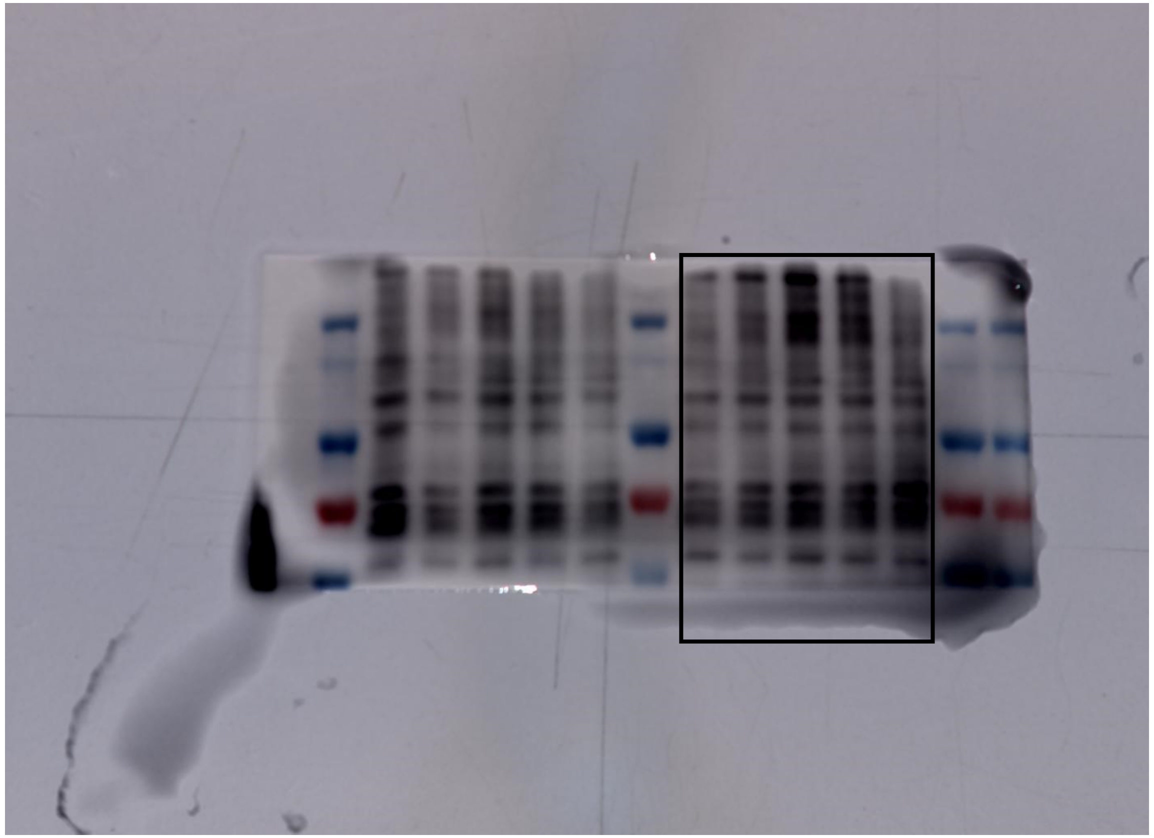

Supplement Figure 2A-β-actin

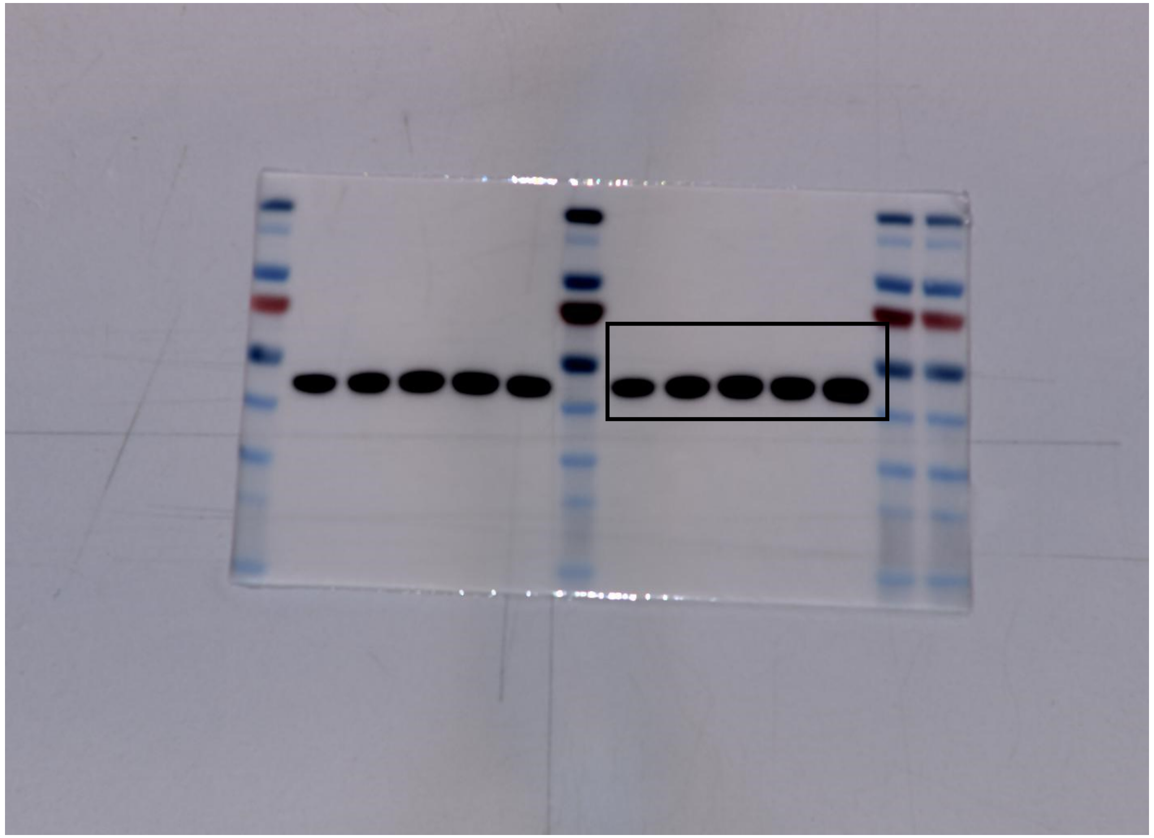

Supplement: Supplementary file 2 — Original full length western blots [file 41420_2024_2114_MOESM2_ESM.pdf]
